# Hulless Black Barley as a Carrier of Probiotics and a Supplement Rich in Phenolics Targeting Against H_2_O_2_-Induced Oxidative Injuries in Human Hepatocarcinoma Cells

**DOI:** 10.3389/fnut.2021.790765

**Published:** 2022-01-28

**Authors:** Han Wu, Hao-Nan Liu, Chun-Quan Liu, Jian-Zhong Zhou, Xiao-Li Liu, Hong-Zhi Zhang

**Affiliations:** ^1^Institute of Agro-Product Processing, Jiangsu Academy of Agricultural Sciences, Nanjing, China; ^2^College of Food Science and Technology, Huazhong Agricultural University, Wuhan, China; ^3^School of Food and Biological Engineering, Jiangsu University, Zhenjiang, China

**Keywords:** black barley, lactic acid bacteria, phenolic transformation, antioxidant activity, human hepatocarcinoma cells

## Abstract

Lactic acid bacteria can provide benefits to human beings and transform phenolic substances to improve their potential functionality. It was of interest to develop black barley as a carrier of probiotics and nutraceutical supplement rich in more antioxidants. Due to fermentation, bacterial counting and free phenolic content in black barley increased to 9.54 ± 0.22 log cfu/mL and 5.61 ± 0.02 mg GAE/mL, respectively. Eleven phenolic compounds, including nine isoflavones and two nitrogenous compounds were characterized using UPLC-QTOF-MS, among which epicatechin, hordatine, and pelargonidin aglycone were largely enriched. Moreover, free phenolic extracts from fermented barley (F-BPE) played a greater role in scavenging DPPH radicals, reducing Fe^3+^ to Fe^2+^, and increasing oxygen radical absorbance capacity, compared phenolic extracts from unfermented barley [UF-BPE (1.94-, 1.71-, and 1.35-fold at maximum for F-BPE vs. UF-BPE, respectively)]. In hepatocarcinoma cells, F-BPE also better inhibited ROS production and improved cell viability, cell membrane integrity, SOD activity, and non-enzymatic antioxidant GSH redox status (2.85-, 3.28-, 2.05-, 6.42-, and 3.99-fold at maximum for F-BPE vs. UF-BPE, respectively).

## Graphical Abstract

Transformation of barley phenolics by fermentation improves their antioxidant capacities in chemical and cell levels. 
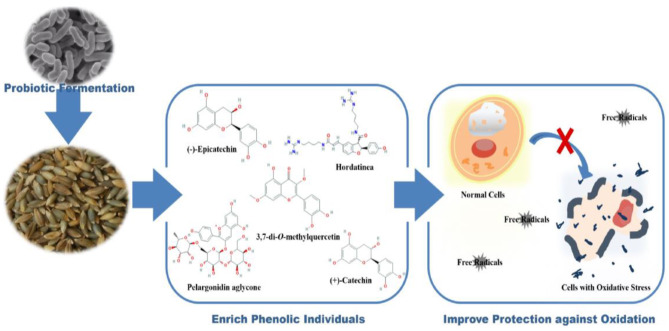

## Highlights

- *Lactobacillus* improved phenolic content and antioxidant activity of black barley.
- UPLC-QTOF-MS analysis was carried out for phenolic compounds from UFB and FB.
- StrainP-S1016 largely enriched epicatechin, hordatine and pelargonidin aglycone.
- FB phenolics better ameliorated oxidative defense system by a composite mechanism.
- A novel strategy was found to develop barley as carrier of probiotics and therapeutic ingredients.

## Introduction

Phenolics are the natural components of plant-based foods and are composed of flavonoids, tannis (hydrolysable and condensed), phenolic acids, stilbenes, lignans, and phenolic aldehydes. As demonstrated in numerous reports, dietary phenolics have some biological effects on scavenging free radicals, regulating digestive enzymes, and chelating metals (1). As previously verified, the optimized ultrasonic-assisted extraction could be used as an eco-friendly technique to better improve the yield and value-adding of phenolic extracts than the stirring-assisted or microwave-assisted extractions (2, 3). Phenolic consumption is considered beneficial for protecting against the oxidation and preventing various chronic diseases, such as ophthalmological, cardiovascular and digestive malfunctions, diabetes, atherosclerosis, allergies, tumorigenesis, and viral infection (4).

Hulless black barley (*Hordeum* spp.) is a special crop that contains valuable sources of minerals, vitamins, and some important bioactive compounds, which grows and matures under arctic–alpine, anoxic, low temperature, and intense ultraviolet conditions. According to epidemiological studies, a negative association exists between metabolic syndrome and the long-term intake of grain foods (5). Commonly, the barley phenolic compounds (PCs) are found in three major forms: soluble free, soluble conjugated that are esterified to sugars and low-molecular-mass components, and insoluble bound states that are covalently bound to structural elements in cell walls, the latter two of which account for the majority of total phenolic content (6). The free PCs can easily be released from plants, whereas the insoluble-bound PCs are not easily extracted due to their interaction with proteins or polysaccharides in cell walls. It is necessary to release the bound phenolics or implement the phenolic transformation before consumption to provide more health advantages to humans (7).

Among different processing techniques, fermentation is one with high specificity, low toxicity, and high performance that facilitates the breaking of covalent bonds and increases the liberation and bioavailability of polyPCs in food materials (8). A presumable mechanism for the fermentation-induced enhancement of phenolic activity in cereal grains is related to the enzymes present in fermenting microbes, causing the transformation of substances, the structural breakdown of cell-wall matrix, and hydrolyzing PCs to separate them from their glycosides or other conjugates (9, 10). Wang et al. (11) confirmed that the leaves of *Psidium guajava* L. had a decreased content of insoluble-bound polyphenol components after being cofermented with *Monascus anka* and *Bacillus* sp., and the soluble PCs exhibited greater protective effects against DNA oxidative damage compared with the insoluble-bound PCs. Similarly, Bei et al. (12) also revealed that the total phenolic content, especially free phenolic content (FPC), was significantly increased by bioprocessing. Considering that lactic acid bacteria (LAB) is generally recognized as safe (GRAS) and possessed the ability to produce various enzymes, such as glycosyl hydrolases, esterases, tannin hydrolases, reductases, and decarboxylases (13), a *Lactobacillus plantarum* strain isolated from plant-based material was used in the present study.

Generally, oxidative stress is manifested by the excessive production of reactive oxygen species and is an insufficient or defective antioxidant defense system involved in aging (1). Until now, many studies have employed fermentation to increase the content of free PCs in cereal products to enhance their antioxidant activity (14, 15). However, only a few researchers paid attention to study the role of microorganisms in improving the antioxidant activity of hulless black barley, and even less, established a cell model to interpret the mechanisms through which the phenolics from fermented black barley protect against the oxidative stress-induced damage. According to Lima et al. (16), HepG2 cells, a human hepatoma cell line, could be adopted as an excellent model to investigate the intracellular mechanisms. H_2_O_2_ is a major factor implicated in the free-radical theory of aging. It can induce oxidative stress and damage biomacromolecules in cells, including nucleic acids, membrane lipids, and proteins (17). On this basis, the influences of fermentation with LAB on the cytoprotection of barley phenolics targeting the H_2_O_2_-induced HepG2 cells were further studied.

In this study, the phenolic transformation of the major individual compounds from the unfermented barley (UFB) to the barley fermented with *L. plantarum* P-S1016 were investigated. The antioxidant capacities of phenolics were then examined using a series of experiments at the chemical and cell levels. Meanwhile, a principal component analysis was performed to learn the correlative relationships among different indexes, including the viable bacterial number, FPC, and different antioxidant variables, and to reveal the effects of fermentation on these indexes. This manuscript focused on the development of hulless black barley as a novel carrier of LAB and nutraceutical supplement rich in antioxidants. The ultimate objective was to extensively realize the comprehensive utilization of hulless black barley and interpret the mechanism by which LAB enhanced the protective effects of PCs against H_2_O_2_-induced oxidative stress in liver cells.

## Materials and Methods

### Materials, Microorganisms, and Chemicals

Black barley was obtained from Jiangsu Coastal Area Institute of Agriculture Sciences, China, and *L. plantarum* P-S1016 was conserved in Jiangsu Academy of Agricultural Sciences, China. Methanol and acetonitrile were purchased from TEDIA (Fairfield, OH, USA) with purifies of > 95.00%. The 2,2-diphenyl-1-picrylhydrazyl (DPPH) and 2,4,6-tris (2-pyridyl)-S-triazine (TPTZ) were purchased from Sigma-Aldrich (St. Louis, MO, USA), 6-hydroxy-2,5,7,8-tetramethylchromate-2-carboxylic acid (Trolox), and 2,2'-azobis (2-methylpropionamide)-dihydrochloride (AAPH) were, respectively, obtained from Acros Organics (Morris Plains, NJ, USA) and J&K Chemical (Beijing, China). A human hepatocarcinoma cell (HepG2) line was obtained from Taide Biological Technology (Nanjing, China). Dulbecco's modified eagle medium (DMEM) and fetal bovine serum (FBS) were obtained from Gibco/Invitrogen (Shanghai, China). MTT cell proliferation and cytotoxicity assay kit, reactive oxygen species (ROS) assay kit, lactate dehydrogenase (LDH) release assay kit, total superoxide dismutase (SOD) assay kit with WST-8, and glutathione (GSH and GSSH) assay kit were purchased from Beyotime Biotechnology (Shanghai, China).

### Inoculum Preparation and Fermentation of Black Barley

The strain was prepared for two successive transfers in de Man-Rogosa and Sharp broth (MRS, pH 6.20 ± 0.20, UK) at 30.0°C for 24.0 h and 12.0 h. The activated microorganisms were collected by centrifugation at 5,000 *g* at 4.0°C for 15.0 min (3K15, Sigma-Laborzentrifugen, Osterode am Harz, Germany) and washed twice with sterilized physiological saline (18, 19). Black barley was dispersed in 5-fold distilled water to make a slurry and sterilized at 121.0°C for 20 min before *L. plantarum* P-S1016 inoculation (3.00%, v/v) at 37.0°C. As previously determined, the fermentation continued for 36.0 h, where the viable counting number had a maximum value. The prepared UFB and fermented barley (FB) were stored at 4.0°C for future use.

### Microbiological Analysis

Viable bacterial count was determined by the plate count method (20). Briefly, for the harvested UFB and FB, 1.00 mL of each sample was homogenized aseptically with 9.00 mL of sterile physiological saline (0.85%, w/w) before a series of 10-fold dilutions. MRS agar (pH 6.20 ± 0.20) was used for bacterial growth at 37.0°C for 48.0 h. The confirmed colonies were counted and their numbers were expressed as log cfu/mL.

### pH Measurement

A total of 20.00 mL of each sample of UFB and FB was fetched and shaken in a blender to measure their pH values with an IS128 pH meter (Insmark Instrument Technology, Shanghai, China) (21).

### Phenolic Extraction

The preparation of phenolic extracts was according to the method of Xiao et al. (15) with a slight modification. Equal weight of the dried samples of UFB and FB of were extracted with 10-fold 80.0% (v/v) ethanol in a water bath at 40.0°C for 3.0 h (DKZ-450B, Sengxin Ultrasonic Instruments, Shanghai, China). The extracted solution was then centrifuged at 10,000 *g* at 4.0°C for 15 min. The supernatant was evaporated to dryness and its substances were reconstituted with distilled water to be lyophilized (Powerdry LL3000, Thermo, Massachusetts, USA). The prepared free unfermented barley phenolic extract (UF-BPE) and fermented barley phenolic extract (F-BPE) powders were sealed and stored at −18.0°C for antioxidant analysis.

### FPC and Insoluble-Bound Phenolic Content Detection

Following the water-bath phenolic extraction, phenolic contents were determined using the Folin–Ciocalteu colorimetric method (22, 23). Briefly, each of the dried samples of UFB and FB weighted 1.00 g, and their relevant supernatants were evaporated, and finally 1.00 mL 80.0% (v/v) ethanol was added to obtain a constant volume. A total of 0.40 mL of each free phenolic solution was oxidized with 2.00 mL 0.50 mol/L Folin–Ciocalteu reagent at room temperature for 4.0 min. Then, the reaction was neutralized by adding 2.00 mL of 75.00 g/L saturated sodium carbonate. After 2.0 h of incubation in the dark, the absorbance at 760 nm was recorded using a spectrophotometer (UV 5500, Metash Instruments, Shanghai, China). The FPC results were expressed as gallic acid equivalent (GAE), i.e., mg GAE/g. Furthermore, the residue from the above extraction was hydrolyzed directly with 50.00 mL of 4.00 M NaOH solution for 4.0 h with shaking. The mixture was adjusted to pH 2.00 with concentrated HCl and extracted with ethylacetate (11). The relevant supernatant was evaporated and finally 1.00 mL of 80.0% (v/v) ethanol was added to a constant volume. The IBPC was measured as above and also expressed as mg GAE/g.

### UPLC-QTOF-MS Analysis

The phenolic extracts were analyzed by HPLC-MS (G2-XS QTOF, Waters Corporation, Massachusetts, USA). Two microliters of phenolic solution was injected into the UPLC column (2.1 × 100 mm ACQUITY UPLC BEH C18 column containing 1.7 μm particles) with a flow rate of 0.35 mL/min. Buffer A consisted of 0.1% formic acid in water and buffer B contained 0.1% formic acid in acetonitrile. The gradient was 5% buffer B for 0.5 min, 5.0–40.0% buffer B over 20.0 min, and 40.0–95.0% buffer B over 2.0 min (24, 25). Mass spectrometry was performed using electrospray source in positive ion mode with MSe continnum acquisition mode, with a selected mass range of 50–1,200 m/z. The capillary voltage was 2.0 kV, collision energy was 10–40 eV, source temperature was 120.0°C, and desolvation gas temperature was 400.0°C.

### *In vitro* Antioxidant Activity Assays

#### DPPH Radical Scavenging Activity Detection

The DPPH radical scavenging activity of black barley extracts (UF-BPE and F-BPE) was determined according to the method of Shimada et al. (26) and Lee et al. (27). Specifically, 2.00 mL of each sample at different concentrations (0–20 μg/mL) was added to 2.00 mL DPPH solution (0.2 mmol/L) previously dissolved in 80.0% (v/v) methanol. The mixture was shaken and allowed to stand in the dark for 30.0 min. The absorbance was then recorded at 517 nm. The activity was calculated as follows: DPPH radical scavenging activity (%) = [1-absorbance of sample/absorbance of control] × 100%.

#### Ferric Reducing Antioxidant Power Detection

The FRAP was evaluated using the method of Benzie and Strain (28) and Qin et al. (29) with slight modifications. A 100.00 mL aliquot of 0.30 mol/L acetate buffer, 10.00 mL of 10.0 mmol/L TPTZ solution in 40.0 mmol/L HCl, and 10.00 mL of 20.0 mmol/L ferric chloride was mixed to prepare the FRAP reagent. A total of 1.00 mL of each sample at different concentrations (0–20 μg/mL) was added to 5.00 mL of FRAP solution, and the mixture was placed in the dark at 37.0°C for 20.0 min. The absorbance was measured at 593 nm. The results were expressed as Fe(II) equivalent antioxidant capacity, i.e., μmol Fe(II)/L, by plotting the standard curve of ferrous sulfate.

#### Oxygen Radical Absorbance Capacity Detection

As for ORAC assay, the reaction was completed at 37.0°C in pH 7.40 phosphate buffer. One hundred microliters of each sample at different concentrations and 50 μL of 0.2 μmol/L fluorescein were mixed in a 96-well microplate. After preincubation at 37.0°C for 15.0 min, 50 μL of 80.0 mmol/L AAPH solution was added immediately. The fluorescence was recorded by an LB 941 TriStar Microplate Reader (Berthold Technologies, Bad Wildbad, Germany) with 485-P excitation and 535-P emission filters every minute during a 100.0-min process (30). The results were expressed as Trolox equivalent antioxidant capacity, i.e., mol Trolox/g.

### Antioxidant Activity Assays in Oxidative-Damaged HepG2 Cells

#### HepG2 Cell Culture and Treatment

HepG2 cells were cultured in DMEM supplemented with 10.00% FBS, 1.00% streptomycin and penicillin, and 1.00% 1.00 mol/L HEPS buffer at 37.0°C in a 5.0% CO_2_ humidified incubator (MIR-254, Sanyo Denki, Shanghai, China) (31). Before the experiment, the cells were quiesced in a reduced serum medium for 4.0 h and then seeded at a concentration of 200,000 cells/mL. According to our preliminary experiment, HepG2 cells were first treated with UF-BPE or F-BPE at different concentrations (1–10 μg/mL) for 6.0 h. Then, 2.40 mmol/L H_2_O_2_ was added to induce oxidative stress and stimulate the cells for 2.0 h. A normal cell group with neither sample pretreatment nor H_2_O_2_ stimulation was used as the control. A group of H_2_O_2_-induced cells was applied as the oxidative model.

#### Cell Viability Measurement

HepG2 cells were cultured according to the treatment above, and cell viability was determined by the MTT method (32). Detailly, the cells were cultured with 20 μL of 5.0 mg/mL MTT for 4.0 h, followed by the removal of the incubation medium. The formazan crystals were dissolved by the addition of 150 μL of dimethyl sulfoxide (DMSO) and slowly shaken for 10.0 min. After the absorbance measurement at 490 nm on an LB 941 TriStar Microplate Reader, the cell viability levels were calculated as follows: viability level (fold increase) = OD value of oxidative-model or sample group/OD value of control group.

#### ROS Detection

A dichlorodihydrofluorescein diacetate (DCFH-DA) detection kit was used to evaluate ROS level in HepG2 cells. The cultured cells were washed with phosphate buffered saline (PBS), and 0.01 mmol/L DCFH-DA was added to each well to start the reaction at 37.0°C for 20.0 min (33). After being washed thoroughly with PBS to remove the DCFH-DA so that it does not enter the cells, the HepG2 cells were collected and suspended in PBS and seeded in the 96-well black plates. The fluorescence was immediately determined by an LB 941 TriStar Microplate Reader with 485-P excitation and 535-P emission filters. The ROS levels of cells were calculated as follows: ROS release level (fold increase) = [cell fluorescence intensity of oxidative-model or sample group/the corresponding cell viability]/[cell fluorescence intensity of control group/the corresponding cell viability].

#### LDH, SOD, GSH, and GSSG Detections

The supernatants of cultured cells with different treatments were collected for LDH activity release analysis, and the lysates of cells were used for SOD activity, and GSH and GSSG content detections. The experiments were all executed using the Assay Kit (Beyotime) and according to the manufacturer's instructions. Firstly, the extracellular and intracellular proteins in each of supernatants and cell sediments were quantified, respectively, using a BCA Protein Assay Kit. Then, as for LDH detection, 10.0% (v/v) LDH releasing agent was added to each well for 1 h before the collection of cells, and then 60 μL of LDH detecting agent, including 2-p-iodophenyl-3-nitropheyltetrazolium chloride (INT) and other chemicals were added to the cell supernatant obtained from each well. After incubation for 30.0 min, the absorbance of each sample was measured at 490 nm (34). LDH release level (fold increase) = LDH activity in protein of the control or oxidative-model or sample group/LDH activity in protein of the release group. The SOD was detected using the WST-8 method. A 20-μL of cell sediment obtained from each well was mixed with 160 μL WST-8/enzyme reagent and 20 μL reaction starter, and incubated for 30.0 min at 37.0°C. The absorbance of each sample was measured at 450 nm (35). SOD release level (fold increase) = SOD activity in protein of the oxidative-model or sample group/SOD activity in protein of the control group. To evaluate the GSH and GSSH levels, the cells were trypsinized, added to deproteinized solution, and inserted into a 96-well plate. A 150-μL of working assay mixture was added in each well for incubation for 5.0 min. Afterward, 50 μL NADPH solutions were also rapidly mixed with each of the reaction products several times. The results were measured with absorbance at 412 nm (36). The GSH and GSSG were expressed as ratios of their contents relative to the corresponding protein contents, i.e., mol/g protein.

### Statistical Analysis

The data was presented as mean ± standard deviation (SD) of five independent experiments. OriginPro^®^ 2021 (OriginLab, Northampton, Massachusetts, USA) was used to plot the figures and calculate the area under fluorescence decay curves. One-way analysis of variance (ANOVA) and Duncan's multiple comparison tests were determined by using IBM SPSS Version 22.0 (SPSS, Chicago, IL, USA). Principal component analysis (PCA) was conducted using SIMCA-P Version 11.5 (Umetrics, Malmö, Sweden). The difference at *p* < 0.05 was considered to be statistically significant.

## Results and Discussion

### Changes in Microbial Growth, Free and Insoluble-Bound Phenolic Contents

**Figure 1:**
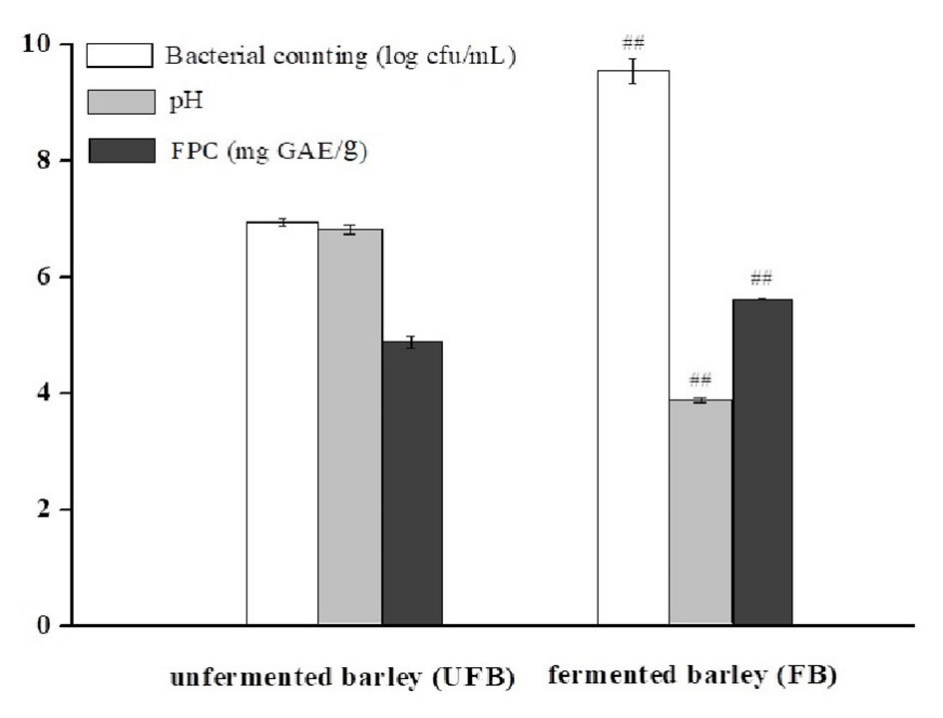
Bacterial counting, pH, and free phenolic content (FPC) of unfermented barley (UFB) and fermented barley (FB). Bars represented mean values ± SD (*n* = 5.00). For the same index, ## indicated significant difference at the level of *P* < 0.01 between UFB and FB.

Figure 1 shows the viable bacterial counts and pH values of UFB and FB samples, respectively. After 36.0 h of fermentation, the LAB population significantly increased from 6.94 ± 0.07 to 9.54 ± 0.22 log cfu/mL (*P* < 0.01). Conversely, the pH value had a downward trend, dropping from 6.78 ± 0.08 to 3.84 ± 0.04 (*P* < 0.01). As shown in Figure 1, the FPC of FB was 5.61 ± 0.02 mg GAE/g, which was significantly higher than that of UFB (4.87 ± 0.01 mg GAE/g) (*p* < 0.01). The content of insoluble-bound phenolic fraction was previously determined as well, and it showed a significant decrease from 3.43 ± 0.01 to 2.16 ± 0.01 mg GAE/g during the whole fermentation process (*p* < 0.01). In this way, the total phenolic content of fermented black barley was slightly improved by approximately 10% compared with that of the unfermented one.

For LAB, the pH decline tendency indicated the growth performance of microorganisms (37). The hydrolytic process of *L. plantarum* P-S1016 enriched the total phenolics and might also lead to the enhancement of cereal bioactivity. It was implied that the increase of total and FPCs were in basic synchronization with the growth of microorganisms. Kaprasob et al. (38) focused on the LAB-based biotransformation of cashew apple juice (CAJ) and also found that *Lactobacillus* spp. improved the TPC in CAJ to a value of 0.13 mg GAE/mL. The results of Wang et al. (11) also showed a significant influence of cofermentation by strater microorganisms on the development of polyphenols from *P. guajava* L. leaves. Consistently, due to fermentation, the total phenolic content in oat increased to 6.04 GAE mg/g, and the FPC in particular, changed with an upward trend (3.27 GAE mg/g) and contributed the majority of the total phenolic content (12). The PCs generally conjugated and insolubly combined with cell wall polymers, such as cellulose, hemicellulose, and lipid moieties through β-glycoside bonds, or other hydrogen and ionic bonds. There might be three reasons why microbial fermentation could increase the content of free phenolics from black barley. One was that the polyphenols in barley mainly existed in the form of bound phenolics, which could be released and transformed into free-form phenolics by alkali, acid, or specific microorganism-derived enzyme system (39, 40). Secondly, microbial metabolism could modify the bioactive substances in barley, leading to the synthesis of new substances such as PCs. Thirdly, *L. plantarum* P-S1016 reduced the pH value and to some extent promoted the release of oat core cell wall degrading enzymes to accelerate the intra-cell compounds releasing (7).

### Identification and Comparison of PCs

The total ion chromatographies of free phenolic extracts, UF-BPE, and F-BPE are presented in Supplementary Figure 1, displaying the apparent amplitude of variation. As detected by UPLC-QTOF-MS, the FB sample contained PCs with more species and more abundance than the UFB sample. Eleven PCs were identified in the black barley. The phenolic identification was conducted by comparing their spectral characteristics in ultraperformance liquid chromatography with diode array detection (UPLC-DAD), and parent and daughter ion information in UPLC-QTOF-MS, with previously reported literature data (Supplementary Figures 2–12) (41–45). According to the classification by KEGG Database (https://www.kegg.jp/kegg/brite.html), these compounds included one proanthocyanidin (*B*-type procyanidin dimer), eight flavonoids (two flavan-3-ols, three flavones, two anthocyanidins, and one flavonol), and two amino-acid-related compounds (*p*-coumaroylagmatine and hordatine A) (Table 1). The flavan-3-ols were composed of (+)-catechin (3.58 min) and (-)-epicatechin (4.97 min), which were two isomerides with [M+H]^+^ at *m/z* 291, and were separated by using the individual standards in UPLC-QTOF-MS detection. The dimer procyanidin *B* ([M+H]^+^ *m/z* 579) involves the monomeric unit of catechin or epicatechin, and thus it produced the dominant product ions at *m/z* 409 and *m/z* 291 upon fragmentation. Among the flavones group, compounds were all in the glycosylated form, which were assigned as isovitexin-7-*O*-glucoside, apigenin-6-C-glucoside-8-C-arabinoside, isoscoparin-2” -*O*-glucoside. Moreover, two anthocyanidins were also confirmed in the colored barley, through comparing the characteristic product ions of anthocyanin aglycones. The peak with [M+H]^+^ at *m/z* 625 was peonidin (*m/z* 301) glycosylated by the sugar moiety of sophorose (*m/z* 324), and that with [M+H]^+^ at *m/z* 625 was pelargonidin (*m/z* 271) conjugated with the sugar moieties of glucose (*m/z* 162) and rutinose (*m/z* 326). The *p*-coumaroylagmatine was with a protonated molecule [M+H]^+^ at *m/z* 277 and MS/MS fragments at *m/z* 217 and 147 [M+H−130]^+^, corresponding to loss of residue agmatine (C_5_H_14_N_4_), and is a member of the class of cinnamamides obtained by formal condensation of the carboxy group of 4-coumaric acid with the primary amino group of agmatine. The hordatine A, a dimer of *p*-coumaroylagmatine, is defined as a phenolamide typical to barley (*Hordeum vulgare*), possessing a critical role in plant development, response to abiotic stress, and defense systems against pathogens and herbivores (41).

**Table 1:** Identification of phenolic compounds in the unfermented and fermented barleys using the spectral characteristic in UPLC and ion fragment information in UPLC-QTOF-MS.

**Compound name**	**Empirical formula**	**RT^a^ (min)**	**λ_max_ (nm)**	**MW^b^**	**[MS-]^c^ (*m/z*)**	**[MS-MS-]^d^ (*m/z*)**	**Change ratio^e^**
*B*-type Procyanidin dimer	C_30_H_26_O_12_	3.25	282	578.50	579	291, 409, 427	−84.39%
(+)-Catechin	C_15_H_14_O_6_	3.58	272	290.27	291	147	+52.64%
*p*-Coumaroylagmatine	C_14_H_20_N_4_O_2_	4.09	290	276.33	277	147, 217	−88.64%
(-)-Epicatechin	C_15_H_14_O_6_	4.97	281	290.27	291	139	+5686.67%
Isovitexin-7-*O*-glucoside	C_27_H_30_O_15_	6.07	328	594.50	595	162, 313, 433	−68.15%
Hordatine A	C_28_H_38_N_8_O_4_	6.20	291	550.70	551	291	+121.83%
Apigenin-6-C-glucoside-8-C-arabinoside	C_26_H_28_O_14_	6.35	329	564.50	565	403, 547	−62.01%
Peonidin-3-*O*-sophoroside	C_28_H_33_${\text{O}}_{16}^{+}$	6.51	520	625.60	625	301	−69.38%
Isoscoparin-2” -*O*-glucoside	C_28_H_32_O_16_	7.60	323	624.50	625	343, 445	−71.39%
Pelargonidin-3-*O*-glucosyl-rutinoside	C_33_H_41_${\text{O}}_{19}^{+}$	8.32	520	741.70	741	271, 433	+88.40%
3,7-Di-*O*-methylquercetin	C_17_H_14_O_7_	14.26	275	330.29	331	301, 315	+70.10%

a *RT was retention time of UPLC-QTOF-MS*.

b *MW was molecular weight of each compound*.

c *[MS-]was mass spectrometry ions*.

d *[MS-MS-] was mass spectrometry-mass spectrometry ions*.

e *Values of change ratio with “–” and “+” indicated the decrease and increase in contents of individual phenolic compounds from black barley due to fermentation with L. plantarum P-S1016, respectively*.

Moreover, the relative contents of individual PCs were compared between unfermented and fermented black barley. The percentages of five compounds were enlarged due to LAB fermentation, including catechin, epicatechin, hordatine, pelargonidin aglycone and 3,7-di-*O*-methylquercetin. Similarly, the aglycones' content in soybean was promoted by *Aspergillus oryzae* and *Monascus purpureus*, as some microorganisms produce key enzymes that destroy the β-glycoside bonds between isoflavones and proteins/polysaccharides in the cell walls in herbal plants (39, 46). The enrichment of certain PCs could also be explained according to others' previous work (41, 47), which indicated that carbohydrate metabolism is the source of the synthesis and transformation of plant secondary metabolites, providing precursors and energy for the subsequent transformation. PCs are a large group of secondary metabolites produced through the phenylpropanoid pathway, where amino acid phenylalanine is the primary starting molecule. As reported, most biosynthesis of aromatic polyketides consist of repeated Claisen condensations of acetyl-coenzyme A (CoA) and malonyl-CoA units, catalyzed by polyketide synthase type. During the biosynthesis of polyketides, polyphenols result from directed cyclocondensations of poly-β-keto intermediates or only partially reduced polyketides chains produced by iterative polyketide synthases (48). Specifically, in the first step of hordatine A biosynthesis, agmatine coumaroyltransferase (ACT) catalyzes agmatine conjugates from pcoumaroyl-CoA, and then the hydroxycinnamic acid agmatines are further oxidatively dimerized to hordatine. The phenolic type and content differences between our unfermented and fermented hulless black barleys might be ascribed to the influence on the substrate environment caused by strain *L. plantarum* P-S1016, and also to the released microbial enzymes acting on the critical points of phenolic metabolite pathways. It was worth mentioning that flavan-3-ols and flavonols possess the conjugated double bonds and multiple hydroxyl groups, attributing to their higher antioxidant activity compared to hydroxycinnamic acids, and the increased anthocyanin not only affects the sensory characteristic and color of barley, but also improves the barley antioxidant activity (49, 50).

### *In vitro* Antioxidant Activities

**Figure 2:**
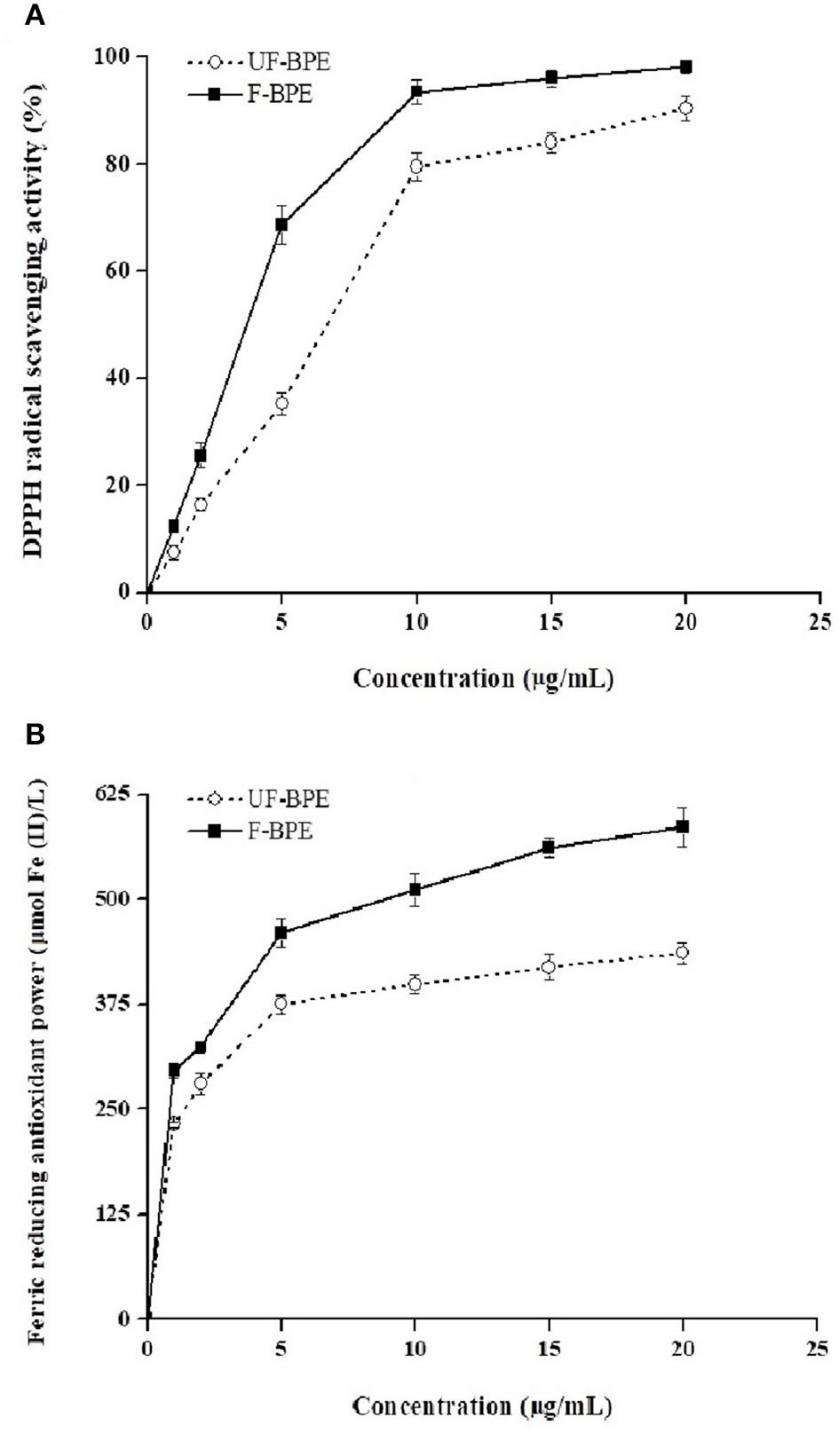
Effects of unfermented barley phenolic extracts (UF-BPE) and fermented barley phenolic extracts (F-BPE) at different concentrations (0.00–20.0 μg/mL) on **(A)** DPPH radical scavenging activity and **(B)** ferric reducing antioxidant power (FRAP). Bars represented mean values ± SD (*n* = 5.00).

Antioxidants are endogenous or exogenous molecules that mitigate any form of oxidative/nitrosative stress of its consequences. According to others' report, the primary protective role of antioxidants in bodies is through their reaction with free radicals (51). In this study, different assays were used to study the antioxidant effects of hulless barley extracts because *in vitro* antioxidant activity should not be determined using a single antioxidant test model. The DPPH radical is a stable lipophilic free radical that is commonly employed to evaluate the free radical scavenging potential of plant extracts (15). Figure 2A showed the dose-response curves for various concentrations of F-BPE and UF-BPE on the DPPH radical scavenging activity. It was observed that the free radical inhibitory activities continued to increase and reached percentages of 90.30% ± 2.34% and 98.15% ± 1.12% for UF-BPE and F-BPE, respectively. At different concentrations (1, 2, 5, 10, 15, 20 μg/mL), F-BPE always performed better in scavenging DPPH radicals than UF-BPE. Here, during the fermentation of barley, the higher antioxidant ability might be attributed to the enrichment in PCs, which reacted with the free radicals and terminated the radical chain reactions (15).

The FRAP had a similar tendency of variation to the DPPH scavenging activity and was enhanced along with the elevated concentrations of phenolic extracts. As Vadivel et al. (52) revealed, the presence of phenolics in extracts caused the reduction of TPTZ-Fe^3+^ complex to TPTZ-Fe^2+^ form and displayed the FRAP. The higher FRAP obtained from the FB might be associated with the release of iron-chelated compounds during fermentation. Figure 2B demonstrated the dose-response curves for various concentrations of F-BPE and UF-BPE on the FRAP. The highest FRAP values for UF-BPE and F-BPE were 436.12 ± 12.01 and 586.02 ± 23.00 μmol Fe(II)/L, respectively. The fermentation process contributed to ameliorating the ferric reducing antioxidant power of barley-derived polyphenols.

Additionally, ORAC may be one of the most suitable methods to assess the *in vitro* antioxidant activity because it utilizes a biologically relevant radical source (1). The ORAC assay was performed to explore the activity of PCs, combining both the time and degree of inhibition. The result indicated that the barley extract's overall antioxidant activity was elevated from 9.66 ± 0.37 to 11.60 ± 0.41 mol Trolox/g (*p* < 0.05) due to fermentation. Our results were consistent with those of others who studied the fermented maize-based product and koji from millet. The studies showed that the general increases in antioxidant activity of fermented foods were attributed to a release of insoluble-bound PC due to activities of hydrolytic enzymes during bioprocessing (53, 54). The discrepancy in antioxidant activities in different studies might be attributed to three significant factors: the microbial strain, the fermentation type, and the fermentation substrate (41). As mentioned above, black barley contained substantial amounts of phenolic antioxidants, and the increased antioxidant activities were positively related with the enrichment in PCs in the FB (55). Due to the potential enhancement of different spectrophotometric antioxidant activities by *L. plantarum* P-S1016 fermentation, a H_2_O_2_-induced oxidative stress in HepG2 cell was further established for in-depth study.

### Antioxidant Activities in Oxidative-Damaged HepG2 Cells

#### Cytotoxicity of Phenolic Extracts in Cells

**Figure 3:**
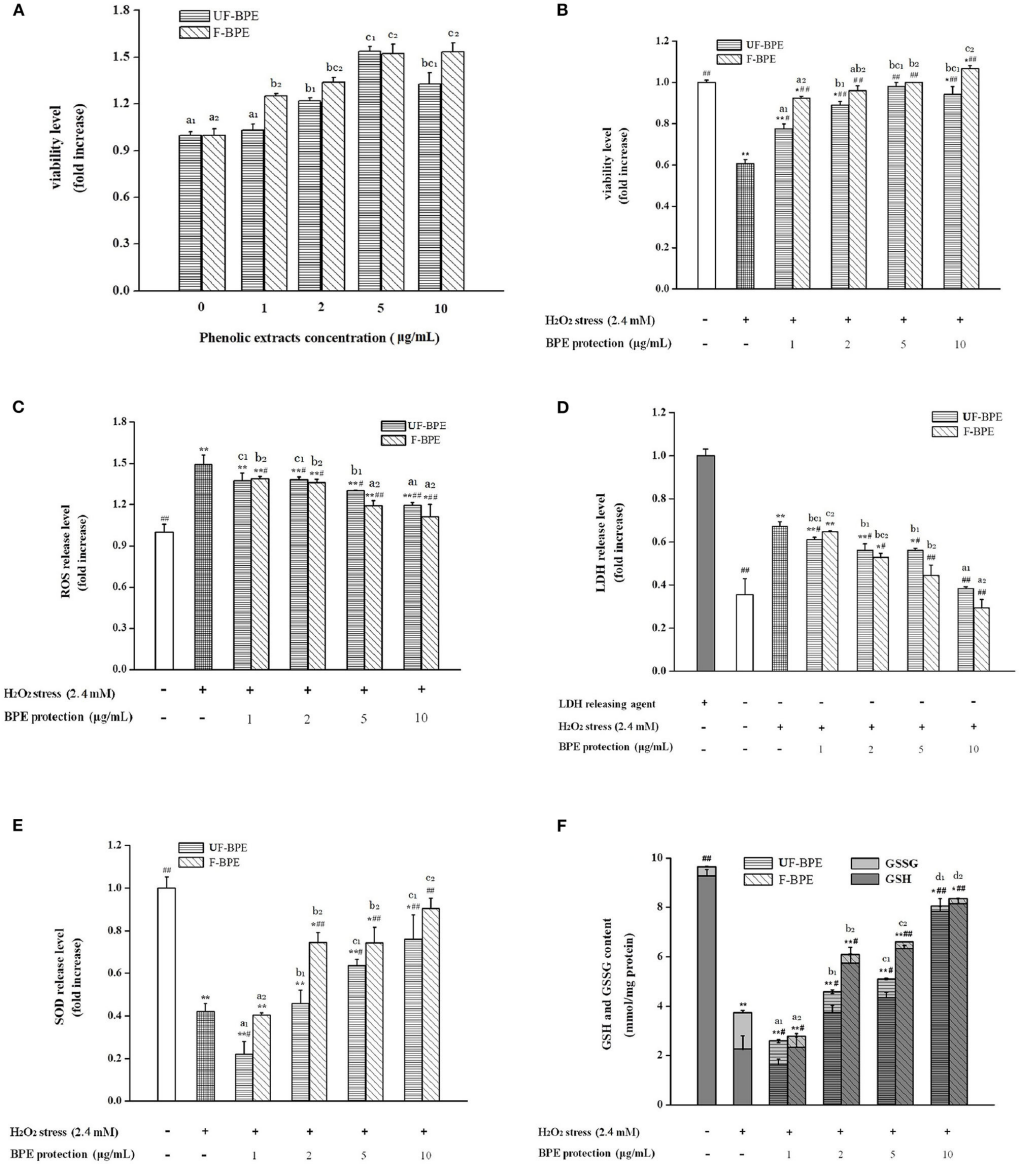
Effects of UF-BPE and F-BPE at different concentrations (1.00–10.0 μg/mL) on **(A)** cytotoxicity, **(B)** cell viability, **(C)** ROS release level, **(D)** LDH release level, **(E)** SOD release level, and **(F)** GSH and GSSG contents in different HepG2 cell groups. Bars represented mean values ± SD (*n* = 5.00). * and ** indicated significant differences at the levels of *P* < 0.05 and *P* < 0.01, respectively, between control group and other groups; # and ## indicated significant differences at the levels of *P* < 0.05 and *P* < 0.01, respectively, between oxidative model group and other groups. Mean values of the same extract with different lowercase letters were significantly different at the level of *P* < 0.05.

To estimate the cytotoxicity of phenolic extracts, they were added at different concentrations in HepG2 cells without H_2_O_2_ stimulation. Compared with the control group, the viability levels of cells pretreated with 1–10 μg/mL extracts ranged between 1.03 ± 0.04- and 1.53 ± 0.06-fold (*p* > 0.05, *P* < 0.01) (Figure 3A). The UF-BPE and F-BPE were proved to have no cytotoxicity on cells and even elevate the cell viability under the normal environment.

#### Protective Effects Against H_2_O_2_-Induced Cytotoxicity, ROS Production in Cells

A potential mechanism by which PCs confer antioxidant activity involves the induction of detoxification mechanisms through phase II conjugation reactions, which prevents the formation of carcinogens from precursors as well as by blocking the reaction of carcinogens with critical cellular macromolecules (56). The phenolics also modify some cellular signaling processes and donate an electron/transfer hydrogen atom to free radicals, activate endogenous antioxidant mechanisms, which increases the levels of antioxidant enzymes, and act as chelators of trace metals involved in free radical protection (57). The oxidative damage should be restricted by intervention of efficient antioxidant-defense mechanisms; however, ROS levels increase and the antioxidant levels decrease in aging tissues (31). In the current study, H_2_O_2_ generated the ROS overproduction, serving as an important biomarker for the assessment of oxidative stress level. As for Figure 3B, it was indicated that pretreatment with UF-BPE and F-BPE (1, 2, 5, 10 μg/mL) for 6.0 h increased the viability of oxidatively injured cells. As for the incubation of UF-BPE prior to H_2_O_2_ induction, the cell viability levels were 0.78 ± 0.02-, 0.89 ± 0.02-, 0.98 ± 0.02- and 0.94 ± 0.04-fold of the control, which were increased by 27.71%, 45.92%, 60.70%, and 54.12% than those in the oxidative model group (*p* < 0.05, *p* < 0.01, *p* < 0.01, *p* < 0.01), respectively. Compared with UF-BPE, F-PBE exhibited a more pronounced ability to reduce the cytotoxic effect of H_2_O_2_ on cells. The cell viabilities of the F-BPE-pretreated groups were 51.21, 57.00, 63.52, and 74.51% greater than those of the oxidative model group (all *P* < 0.01). Consistently, this result demonstrated that *L. plantarum* P-S1016 enhanced the antioxidant activity of barley phenolics.

The fluorescence probe DCFH-DA was used to detect the intracellular ROS level. Here in Figure 3C, H_2_O_2_ significantly increased the ROS level in HepG2 cells by 49.20% (*p* < 0.01 vs. the control group), whereas incubation with phenolic extracts effectively decreased the H_2_O_2_-induced ROS level. Moreover, there existed a dose–effect relationship between the phenolic concentrations and the ROS release levels. When the concentrations of BPE were 1, 2, 5, and 10 μg/mL, the production of H_2_O_2_-induced ROS was significantly inhibited by 7.98, 7.51, 12.71, and 19.95%, respectively, for UF-BPE (*p* > 0.05, *p* < 0.05, *p* < 0.05, *p* < 0.01 vs. the oxidative model group), and 7.04, 8.98, 20.20, and 25.51%, respectively, for F-BPE (*p* < 0.05, *p* < 0.05, *p* < 0.01, *p* < 0.01 vs. the oxidative model group). Our results indicated that the PCs from FB had a better performance in impairing the overproduction of ROS in cells under oxidative stress. As previously reported, ROS had the destructive actions on both proteins and DNA and are therefore regarded in pathogenesis, resulting in cellular death and arterial disease (58). The reduction in ROS was relevant to the amelioration of some side effects, such as DNA mutation and genetic instability. Thus, the fermentation of black barley with *L. plantarum* P-S1016 improved the antioxidant activity of barley.

#### Effects on LDH and SOD Activities in Cells

Moreover, when cell apoptosis or necrosis occurs, the cytoplasmic enzyme LDH is released rapidly following plasma membrane damage. The LSG leakage assay is usually utilized to indicate the cell membrane's integrity for further assessing the cell damage caused by oxidative stress (59). SOD is another antioxidant enzyme, which catalyzes the transformation of toxic superoxide radicals into ordinary molecular oxygen or hydrogen peroxide and plays an important role in cell protection in response to H_2_O_2_-induced oxidative stress (31). In this study, triton X-100 was used as the releasing agent, and the activity level of LDH completely released was considered one (Figure 3D). After exposure to 2.40 mmol/L H_2_O_2_, LDH activity in the cell culture supernatant increased from 0.36 ± 0.07- to 0.67 ± 0.02-fold (1.86-fold, *P* < 0.01 vs. the control group). The higher LDH release level suggested that serious cell injury was generated. However, UF-PBE and F-PBE attenuated LDH release in a positive concentration-dependent manner. The inhibitory rates for UF-BPE and F-BPE (2–10 μg/mL) were 16.50–42.91% and 21.46–56.42% (*p* < 0.05 or *p* < 0.01 vs. the oxidative model group), respectively. For the F-BPE at concentrations of 8 and 10 μg/mL, the LDH release levels were not significantly different from those of the control group (*p* > 0.05). The LDH activities in supernatants of BPE-protected cells were greatly decreased compared with those in the H_2_O_2_-induced oxidative model. Consistently, F-PBE displayed a better capacity to repair the injured cells and protect cells from oxidative damage induced by H_2_O_2_.

As shown in Figure 3E, the SOD activity in cells stimulated by H_2_O_2_ for 2.0 h was significantly reduced (0.42 ± 0.04-fold, *p* < 0.01 vs. the control group), probably related to the dramatic decrease in both viability and LDH release in cells induced by H_2_O_2_. Moreover, BPEs at different concentrations possessed diverse abilities to improve the intracellular SOD levels. UF-BPE (5 and 10 μg/mL) significantly increased SOD activities by 51.02 and 80.70%, respectively (*p* < 0.05 and *p* < 0.01 vs. the oxidative model group). F-BPE showed a more pronounced influence on protection against oxidative stress in H_2_O_2_-treated HepG2 cells. F-BPEs at different concentrations (2, 5, and 10 μg/mL) significantly promoted the production of SOD in a dose-dependent manner, and correspondingly, the SOD activities were augmented by 77.21, 76.78, and 115.02% (all *p* < 0.01 vs. the oxidative model group). Due to the higher SOD activities exhibited in cells of the BPE-treated groups than those of the oxidative model group, phenolics, particularly from barley fermented with *L. plantarum* P-S1016, defensed against the cellular oxidative damage.

#### Effects on GSH and GSSH Contents in Cells

As an important intracellular non-enzymatic antioxidant, glutathione is the most abundant non-protein thiol in eukaryotic cells. Reduced glutathione (GSH) usually served as an electron donor to detoxify endogenous peroxides, leading to the formation of oxidized glutathione (GSSG) (31). The glutathione redox status level, namely, the GSH/GSSG ratio, might be also a critical indicator of oxidative stress in cells and organisms (60). Contents of GSH and GSSG were measured to assess the change in glutathione redox status in HepG2 cells with different BPE treatments. As indicated, glutathione existed mainly in the reduced form in the control group cells, where GSH accounted for 96.20% of the total (Figure 3F). The induction of H_2_O_2_ for 2.0 h caused a reduction in the total contents of GSH and GSSG in cells, and a distinct reversal occurred. The GSH content decreased from 9.29 ± 0.25 to 2.26 ± 0.53 mmol/mg protein and that of GSSG was raised from 0.37 ± 0.02 to 1.47 ± 0.09 mmol/mg protein. The decline in GSH/GSSG ratio clearly indicated a cellular peroxide dominant status. However, Figure 3F also showed that the phenolic extracts from barley induced the regeneration of GSH from GSSG under H_2_O_2_-induced condition. With the protection of BPE (2, 5, and 10 μg/mL), the total glutathione content was significantly higher than that in the oxidative model group (*p* < 0.05 or *p* < 0.01), and the GSH/GSSG ratio in cells was augmented progressively. As for UF-BPE and F-BPE treatments, the GSHs were determined to be 0.65–2.46- and 1.54–2.61-fold higher, respectively (*p* < 0.05 or *p* < 0.01 vs. the oxidative model group), whereas the GSSG was notably lost by 0.34–0.85- and 0.69–0.86-fold, respectively (*p* < 0.05 or *p* < 0.01 vs. the oxidative model group). These findings declared that BPE turned the cellular environment into that with a relatively lower degree of oxidative stress. Compared with UF-PBE, F-PBE displayed an enhanced performance in GSH production and peroxide inhibition, consequently supporting its more protective effects on H_2_O_2_-damaged HepG2 cells.

Overall, the barley extracts exhibited positive effects on cell oxidative defense system through enzymatic and non-enzymatic mechanisms. Compared with UF-BPE, F-BPE was better at increasing cell viability and membrane integrity, scavenging ROS, enhancing the activity level of the key intracellular antioxidant enzyme SOD, and the status of glutathione redox system. Our results were consistent with that reported by Granado-Serrano et al. (61), who found that quercetin contributed to preventing potential oxidation in HepG2 cells and the liver. Also, microorganisms were helpful for boosting the potent activity of PCs from mulberry and blackberry to protect cells against oxidative injury and facilitate cell growth or proliferation again (62). The F-BPE showed more advantages in resisting oxidation than UF-BPE in both the chemical tests and the HepG2 cells. The transformed phenolics from fermented black barley were able to protect cells more effectively against H_2_O_2_-induced damage by a composite antioxidant mechanism.

### PCA Analysis

**Figure 4:**
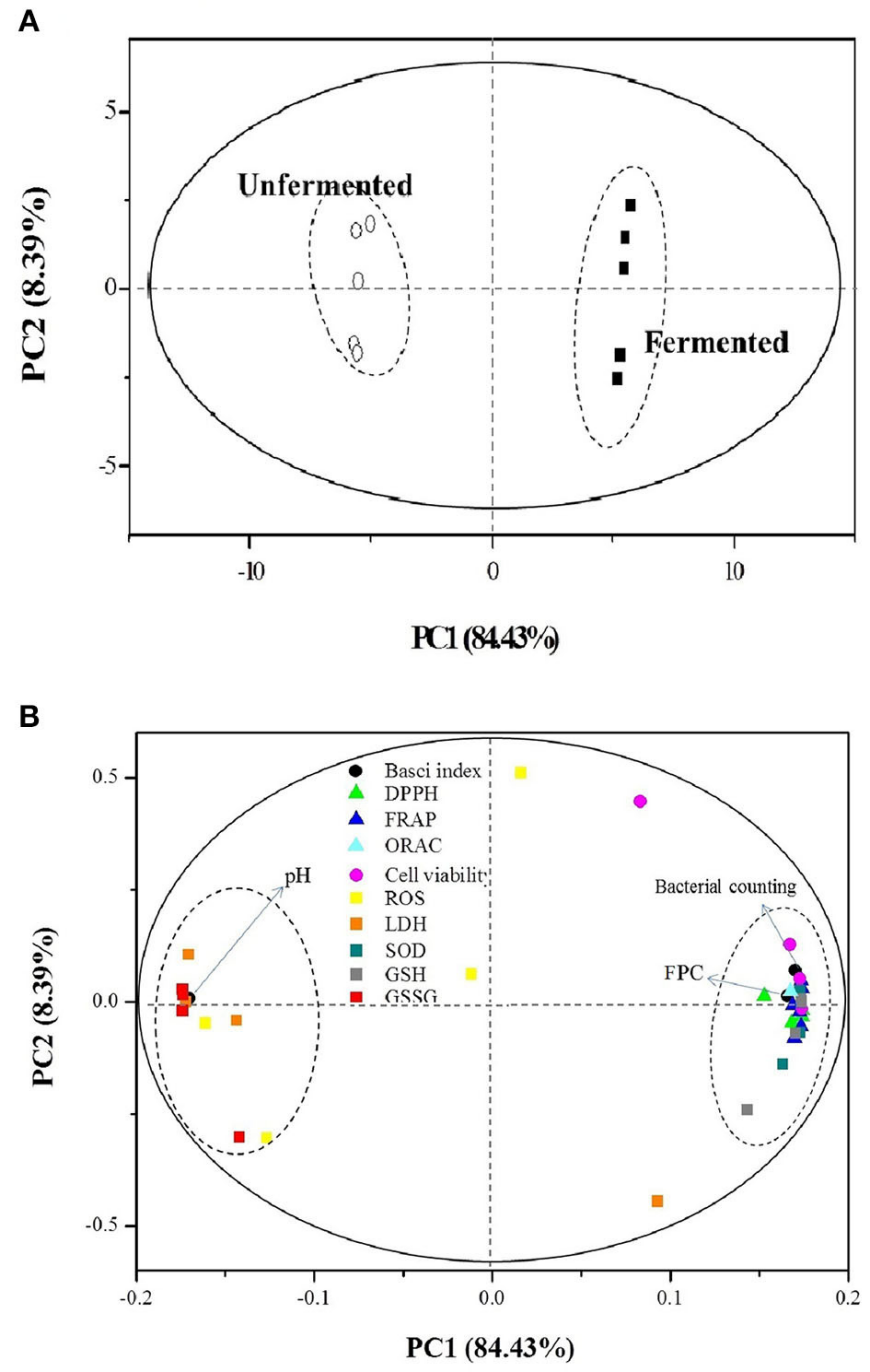
Plots of **(A)** sample scores and **(B)** parameter loadings of principal component analysis (PCA) involving 12 variables on UF-BPE and F-BPE.

The PCA provided valuable information on LAB fermentation's influence on various parameters, especially on the antioxidant capacities. There were two principal components (PC1 and PC2), clarifying up to 92.83% of the total variance. The score plot (Figure 4A) showed that the unfermented and fermented samples were distributed on the left (PC1 <0.00) and right (PC1 > 0.00), respectively. The converse dimension indicated that *L. plantarum* P-S1016 fermentation could make a noticeable difference in barley, taking into account their antioxidant activities. Moreover, Figure 4B divided the 12 parameters into two categories. The bacterial counting, FPC, DPPH, FRAP, ORAC, cell viability under BPE protection, SOD, and GSH were well correlated to each other according to their high loadings on PC1, which reflected the mutual relevance between FPC and other indexes involving the antioxidant activities, antioxidant enzyme, and non-enzymatic antioxidant in cells. The results demonstrated that the PCs from black barley could modify some cellular signaling processes and donate an electron/transfer hydrogen atom to free radicals, activate endogenous antioxidant mechanisms, thus increasing the levels of antioxidant enzymes and acting as chelators of trace metals involved in free radical protection (63). Consistent with the results related to antioxidant performance of barley extracts, PCA also revealed that fermentation by *Lactobacillus plantarum* was an effective tool to produce barley rich in phenolics possessing superior antioxidant activity.

As we all know, to exert the health benefits, PCs need be bioavailable. One key step in bioavailability is bioaccessibility: the release of bioactive compounds in the gastro-intestinal tract and the solubility of these compounds in the digestive fluid which could be determined by *in vitro* digestion (64). Once the phenolics are released and dissolved in intestinal juices, they are potentially accessible for absorption by the epithelial cells of the intestinal wall into the blood stream and available for use. However, these compounds are susceptible to binding with other diet elements released during digestion, like minerals, carbohydrates, dietary fiber, or proteins that affect the solubility and bioaccessibility of PCs (65). If the phenolics bind with other components to form substances that have large molecular masses under the relatively high pH in the intestinal tract, such molecules cannot pass through the dialysis tube and lead to a lower bioavailability (66).

Compared with the soluble free phenolics, the insoluble-bound phenolics are commonly of lower bioaccessibility during digestion (67). Corona-Leo et al. (68) found that the recovery and bioaccessibility index of FPC in apples ranged from 7.23 to 26.61%, and 20.79 to 81.43%, respectively for the gastric and intestinal steps (68). Gong et al. (69) also reported that the FPCs of digested cereal grains ranged from 2.89 (wheat) to 4.23 (corn). In our study, the microbial fermentation resulted in a variation of FPC from 4.87 mg GAE/g to 5.61 mg GAE/g, which was consistent with these above reports. The release and solubilization of phenolics from black barley by microbial enzymes in our study could further take advantage of the potential health benefits of phenolics, as only those phenolics that are soluble can be potentially bioaccessible.

It was found that the phenolic acids in cereals and the products of cereal had a very low bioavailability of substance in human body, with figures as low as 3% (70). Mateo Anson et al. (71) demonstrated that in a dynamic *in vitro* system, the bioavailability of phenolics from bran and aleurone was lower than 1%, assuming that the sole free fraction is a significant contributor. Recently, the Dietary Guidelines for Americans recommended a consumption of at least 3 ounce equivalents of whole meal cereals per day for an adult (72). In this case, it was hypothesized that our black barley-based fermented product could contribute to approximately more than 10 ng/g PCs that are ultimately absorbed after the dietary intake of product. This result was in accordance with others' findings obtained by using *in vivo* experiments. For example, the anthocyanins, representative substance of the PCs, have been confirmed to have protective effects against H_2_O_2_-induced oxidative injury in human cells, and their accumulated concentration was detected to be 0.709 ng/g in pigs and 115 ng/g in rats, respectively (73).

## Conclusions

The present study investigated the biotransformation of black barley phenolics and evaluated the amelioration of antioxidant activity of phenolics at the chemical and cellular levels. After fermentation, the bacterial counting and the total and FPCs of barley considerably increased. The phenolics from FB performed better on chromatographic profile than those from UFB. Eleven PCs were then identified and quantitatively compared, among which two flavan-3-ols, one flavonol, hordatine A, and pelargonidin-3-*O*-glucosyl-rutinoside were enriched by fermentation. Furthermore, F-BPE possessed better activity in scavenging DPPH radicals and improving FRAP and ORAC, and also had a more remarkable influence on ameliorating the oxidative defense system in HepG2 cells. As concluded, *Lactobacillus* accelerated the release of barley phenolics accessible for absorption, improved their phenolic composition, and largely enriched the compounds such as epicatechin, pelargonidin aglycone, etc. The cytoprotective effects of phenolics were therefore enhanced and performed through a composite mechanism, attributing to the fermentation by *Lactobacillus*. LAB fermentation could be considered a novel approach to develop barley as a source of cereal-derived therapeutic ingredients. In the next study, the recovery and bioacccessibility of phenolics from this black barley-based fermented product should be further investigated, and the bioavailability of phenolics and their health benefits would be studied *in vivo* as well.

## Data Availability Statement

The original contributions presented in the study are included in the article/Supplementary Material, further inquiries can be directed to the corresponding author/s.

## Author Contributions

Conceptualization was done by HW and X-LL. Data curation was done by HW and C-QL. Formal analysis was done by H-NL and C-QL. Funding acquisition was done by HW, X-LL, and H-ZZ. HW and H-NL performed the investigation. The methodology was given by J-ZZ, the project administration by HW, the resources by X-LL, and software by J-ZZ. The supervision was done by X-LL and validation by HW. Visualization was done by HW and C-QL, writing the original draft by HW, and writing-review and editing by HW, X-LL, and H-ZZ. All authors contributed to the article and approved the submitted version.

## Funding

This work was financially supported by the Science and Technology Plan Program of Yangzhou City (Grant No. YZ2020045) and Institute Research Fund of Jiangsu Academy of Agricultural Sciences (Grant No. 013036611703).

## Conflict of Interest

The authors declare that the research was conducted in the absence of any commercial or financial relationships that could be construed as a potential conflict of interest.

## Publisher's Note

All claims expressed in this article are solely those of the authors and do not necessarily represent those of their affiliated organizations, or those of the publisher, the editors and the reviewers. Any product that may be evaluated in this article, or claim that may be made by its manufacturer, is not guaranteed or endorsed by the publisher.
