# Supplementary material for: Hulless Black Barley as a Carrier of Probiotics and a Supplement Rich in Phenolics Targeting Against H2O2-Induced Oxidative Injuries in Human Hepatocarcinoma Cells
Source: Front Nutr. 2022 Jan 28;8:790765. doi: 10.3389/fnut.2021.790765 (PMC8833231; doi:10.3389/fnut.2021.790765)

List of figures

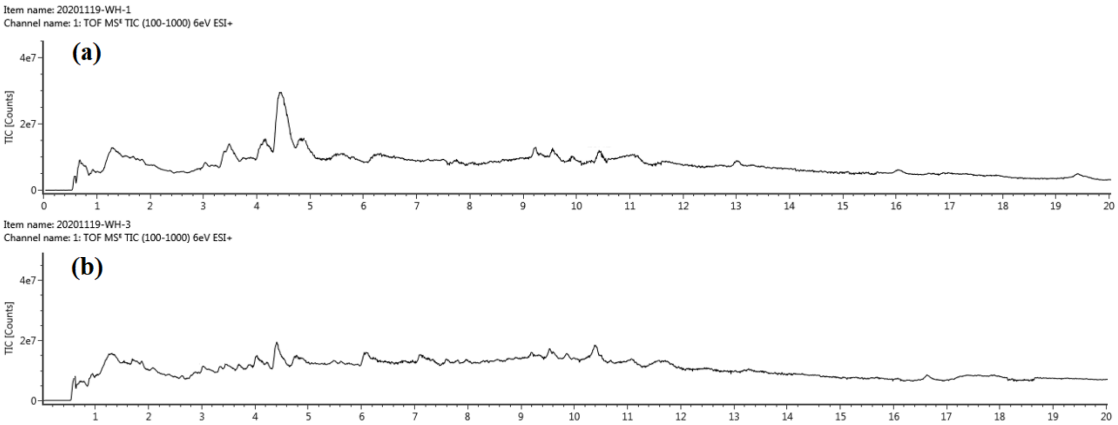

Supplementary Fig. 1 Total ion chromatography of phenolic extracts from unfermented barley (a) and fermented barley (b).

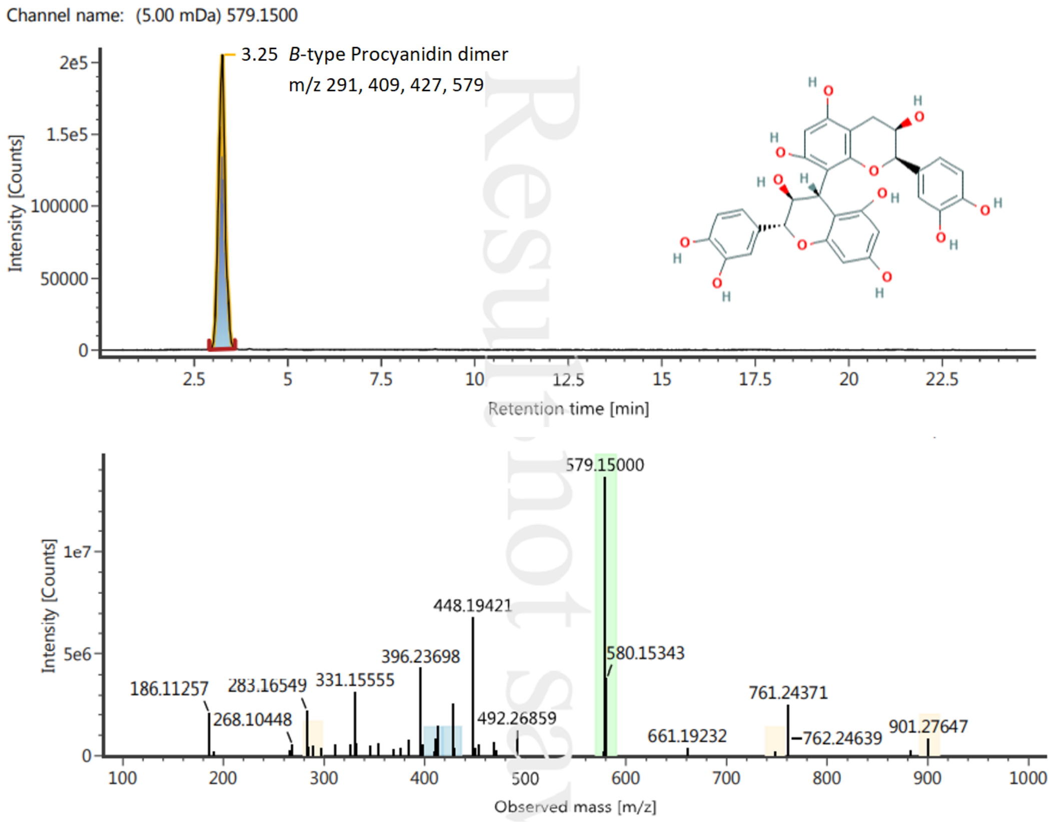

.	Expected m/z	Status	Observed m/z	Mass error (ppm)	Mass error (mDa)	Detector counts	Observed RT (min)	Formula	Observed ion ratio
1	163.03897	Theoretical	163.03911	0.84	0.1	4836	3.23	C9H7O3	
2	247.06010	Theoretical	247.06033	0.94	0.2	10495	3.23	C13H11O5	
3	271.06010	Theoretical	271.06000	-0.36	-0.1	8686	3.24	C15H11O5	
4	275.05501	Theoretical	275.05510	0.32	0.1	8082	3.23	C14H11O6	
5	287.05501	Theoretical	287.05493	-0.28	-0.1	18229	3.24	C15H11O6	
6	289.07066	Theoretical	289.07039	-0.94	-0.3	21553	3.24	C15H13O6	
7	291.08631	Theoretical	291.08545	-2.96	-0.9	14165	3.23	C15H15O6	
8	299.05501	Theoretical	299.05441	-2.01	-0.6	3908	3.23	C16H11O6	
9	301.07066	Theoretical	301.07049	-0.57	-0.2	10900	3.24	C16H13O6	
10	409.09179	Theoretical	409.09119	-1.46	-0.6	41814	3.24	C22H17O8	
11	427.10236	Theoretical	427.10169	-1.57	-0.7	26272	3.24	C22H19O9	

Supplementary Fig. 2 MS of phenolic compound *B*-type Procyanidin dimer. The mother ion (579 m/z) and fragment ions (291, 409, 427 m/z) obtained were consistent with https://mona.fiehnlab.ucdavis.edu/spectra/display/PM004104, which was as follows:

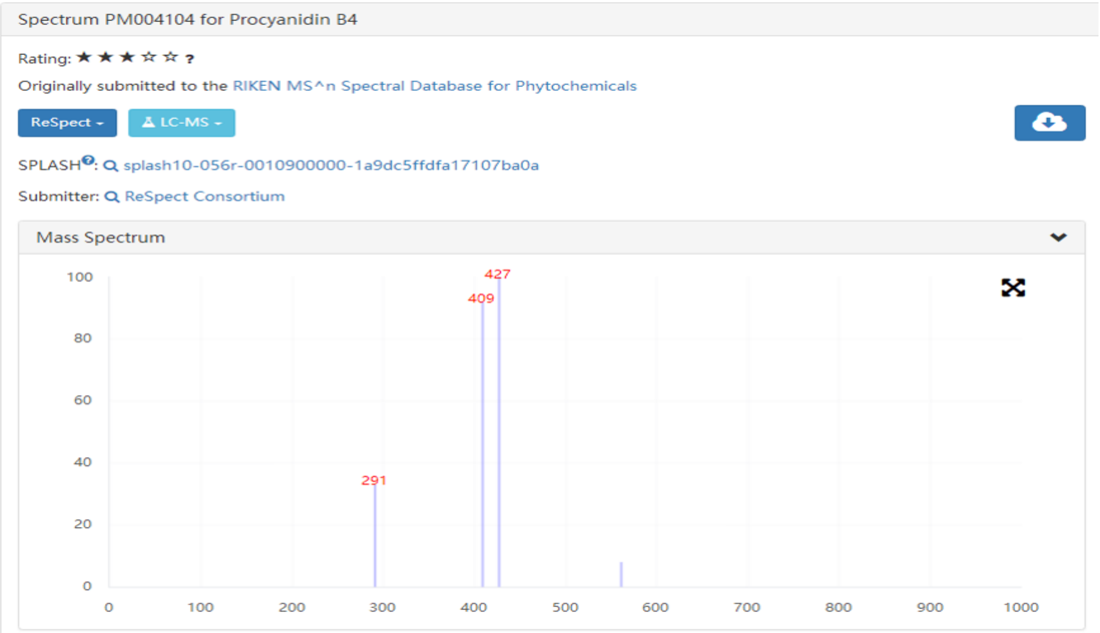

The MS response (3.25 min, m/z 579) of phenolic extracts from unfermented barley (WH-1) and fermented barley (WH-3) were further obtained as follows:

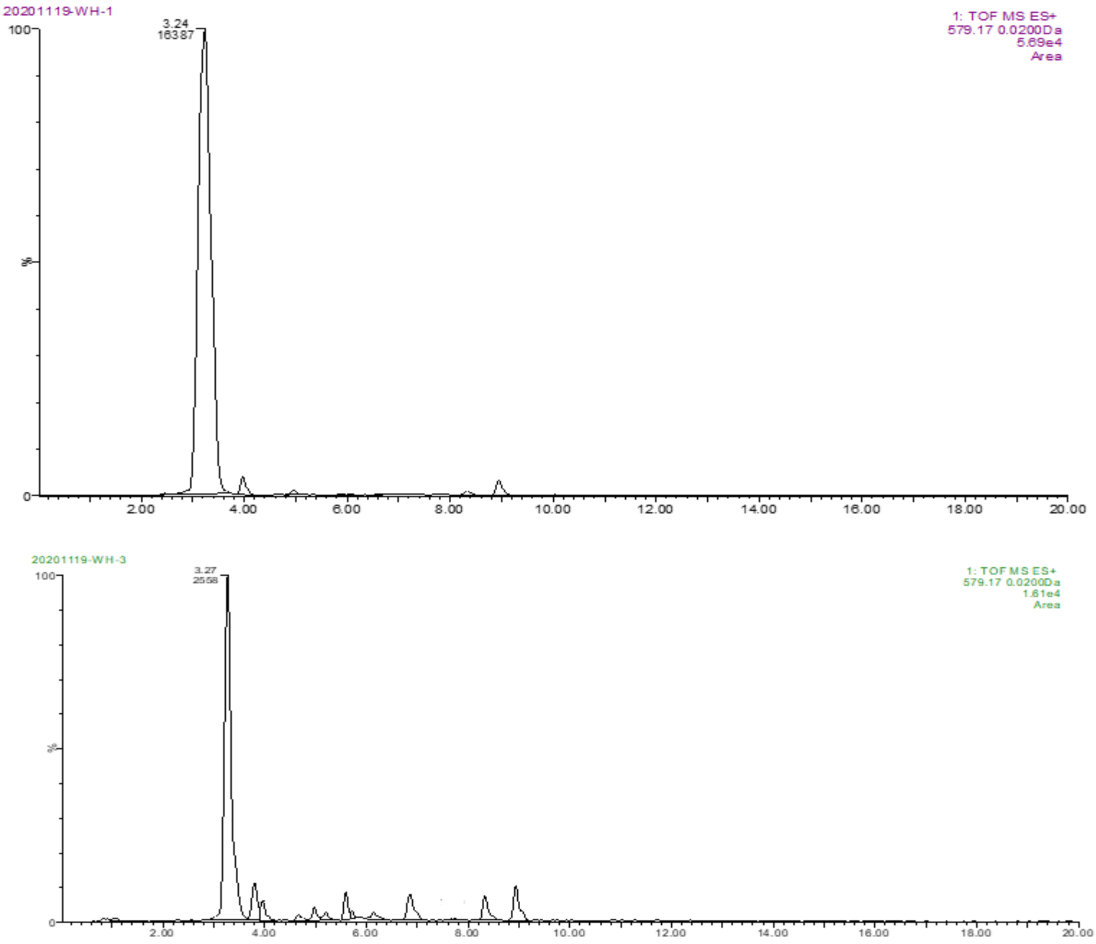

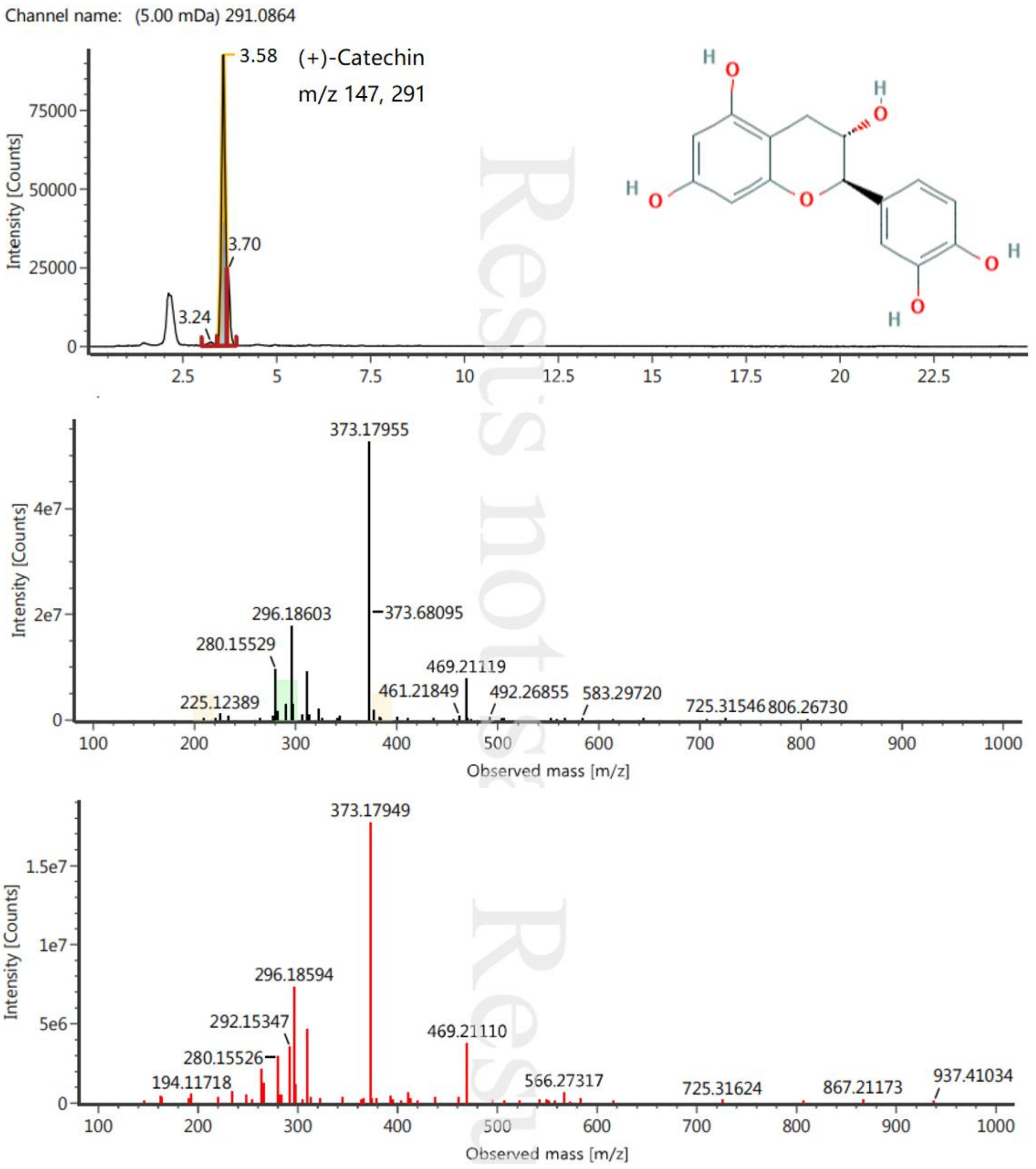

.	Expected m/z	Status	Observed m/z	Mass error (ppm)	Mass error (mDa)	Detector counts	Observed RT (min)	Formula	Observed ion ratio
1	147.04406	Theoretical	147.04402	-0.24	0.0	2182	3.59	C9H7O2	

Supplementary Fig. 3 MS of phenolic compound (+)-Catechin. The mother ion (291 m/z) and fragment ion (147 m/z) obtained were consistent with https://mona.fiehnlab.ucdavis.edu/spectra/display/CCMSLIB00000081476, which was as follows:

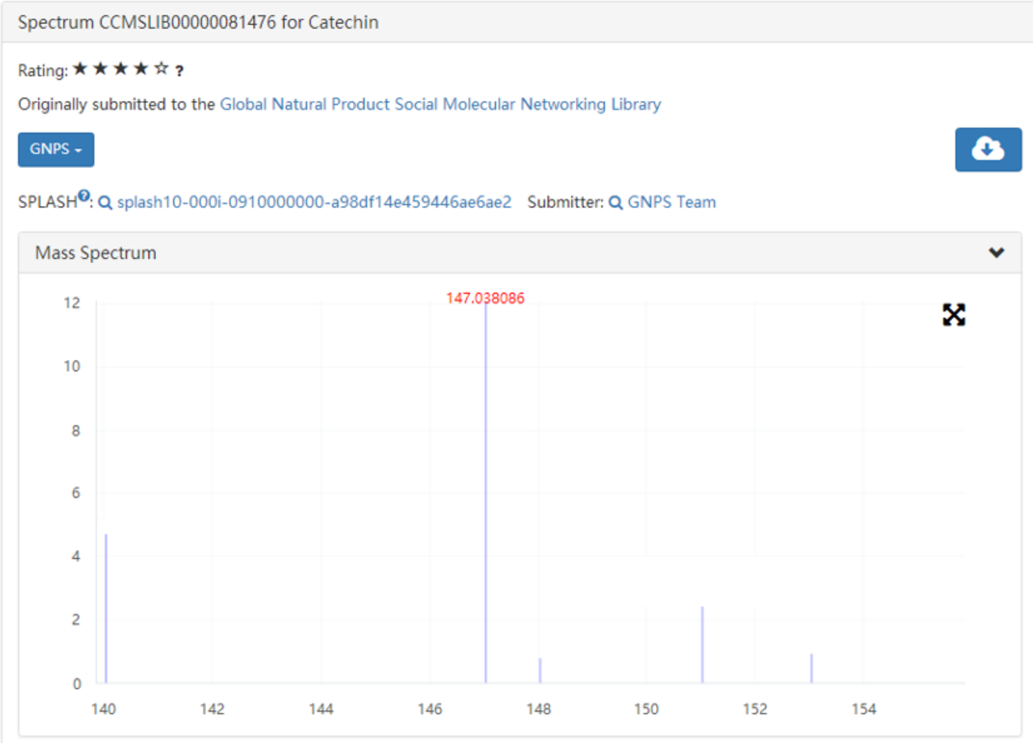

The MS response (3.58 min, m/z 291) of phenolic extracts from unfermented barley (WH-1) and fermented barley (WH-3) were further obtained as follows:

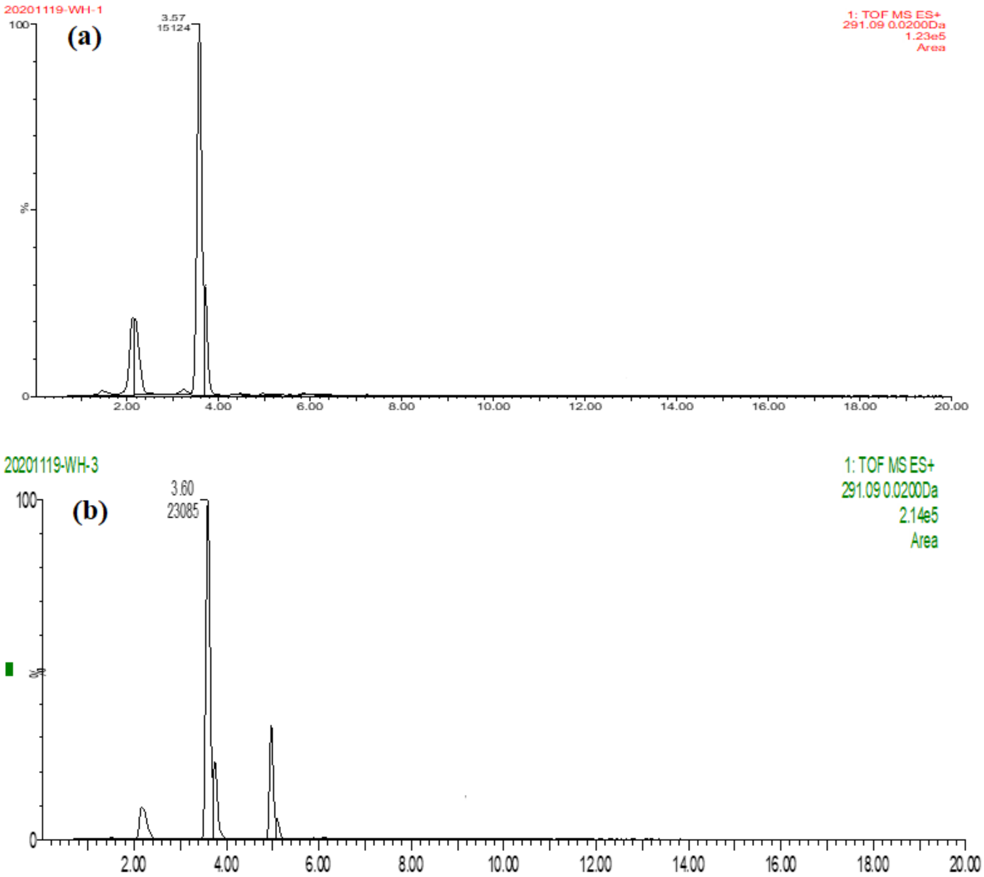

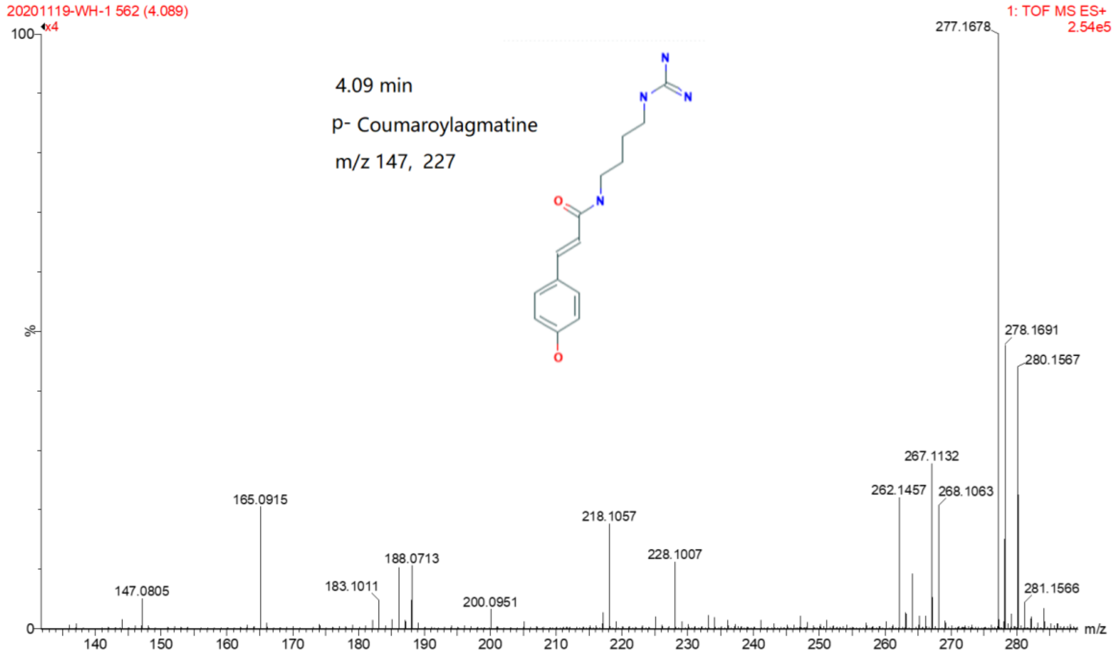

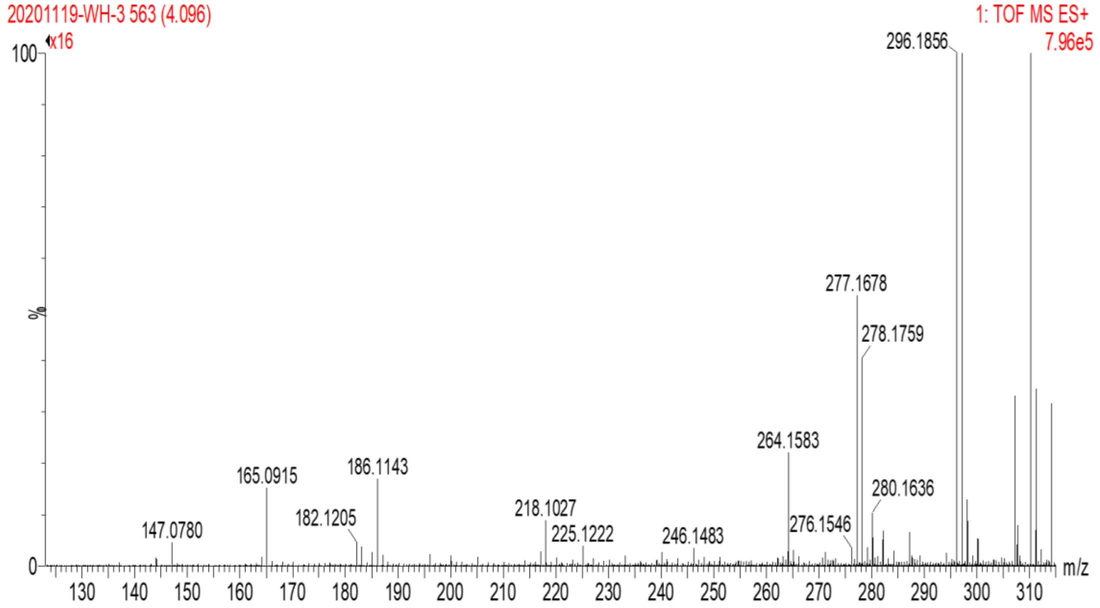

Supplementary Fig. 4 MS of phenolic compound p-Coumaroylagmatine. The mother ion (277 m/z) and fragment ion (147 m/z) obtained were consistent with https://mona.fiehnlab.ucdavis.edu/spectra/display/PM018186, which was as follows:

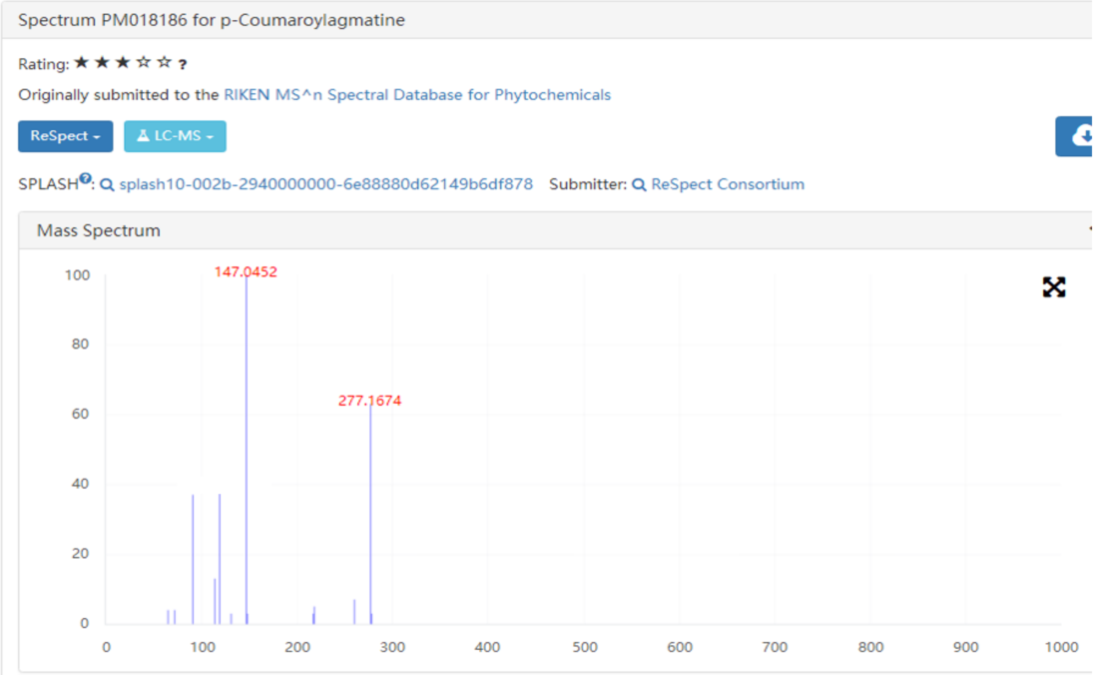

The MS response (4.09 min, m/z 277) of phenolic extracts from unfermented barley (WH-1) and fermented barley (WH-3) were further obtained as follows:

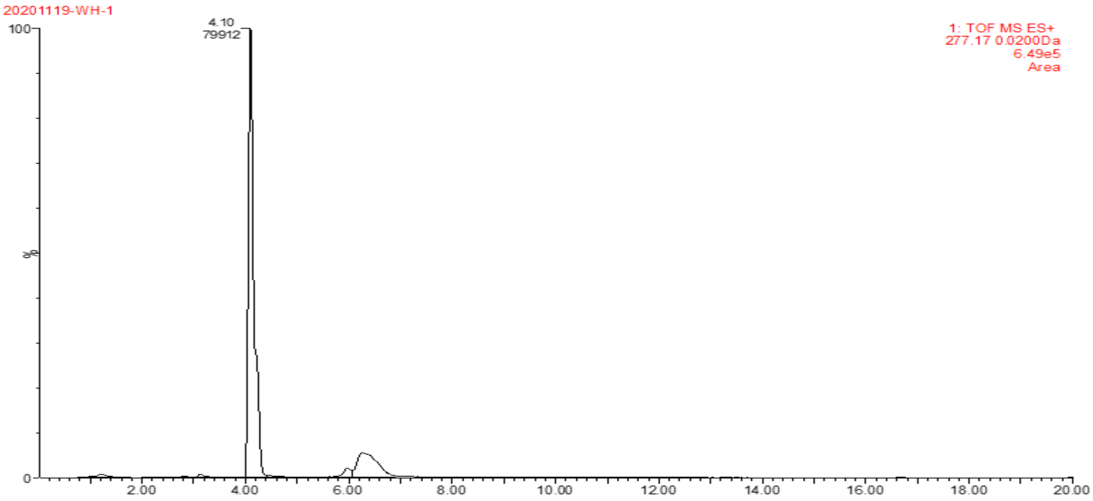

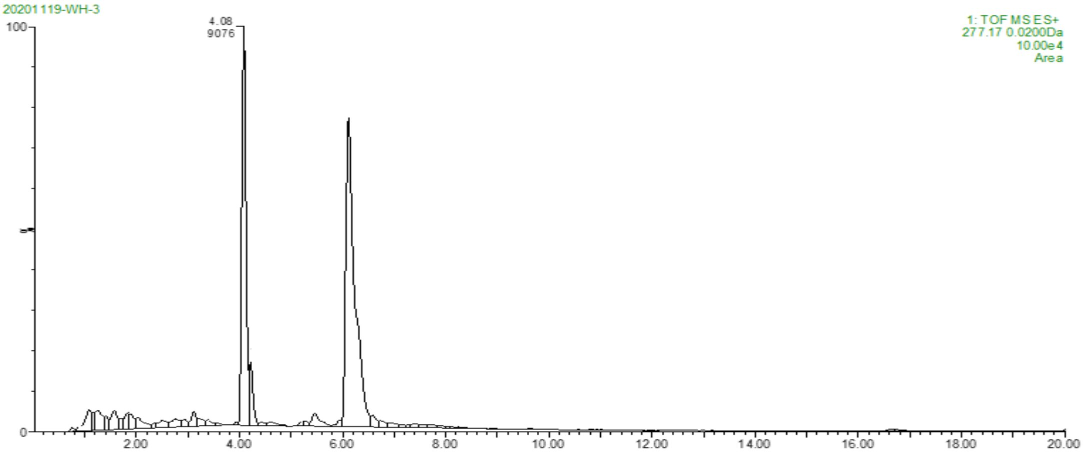

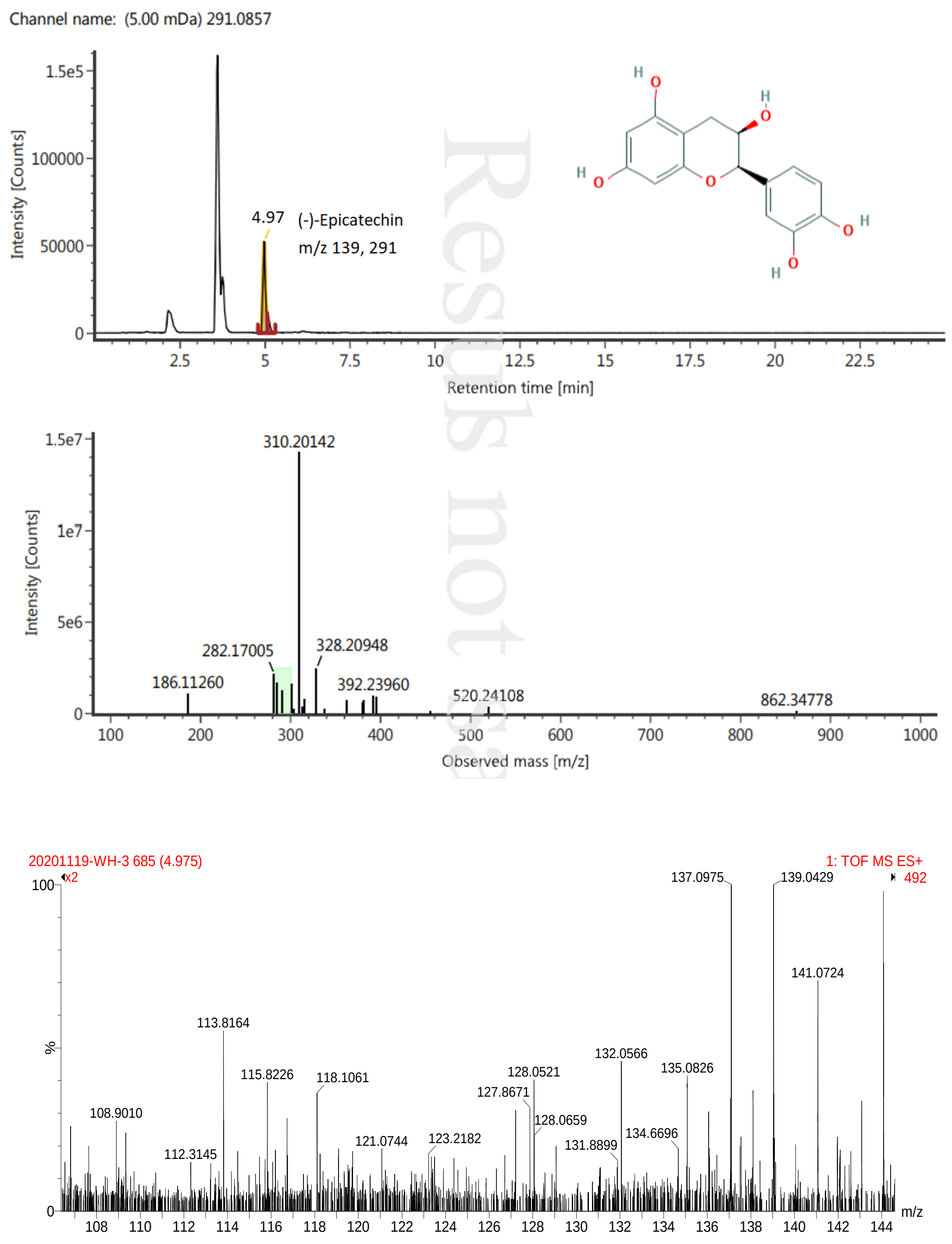

Supplementary Fig. 5 MS of phenolic compound (-)-Epicatechin. The mother ion (291 m/z) and fragment ion (139 m/z) obtained were consistent with https://mona.fiehnlab.ucdavis.edu/spectra/display/FiehnHILIC001913, which was as follows:

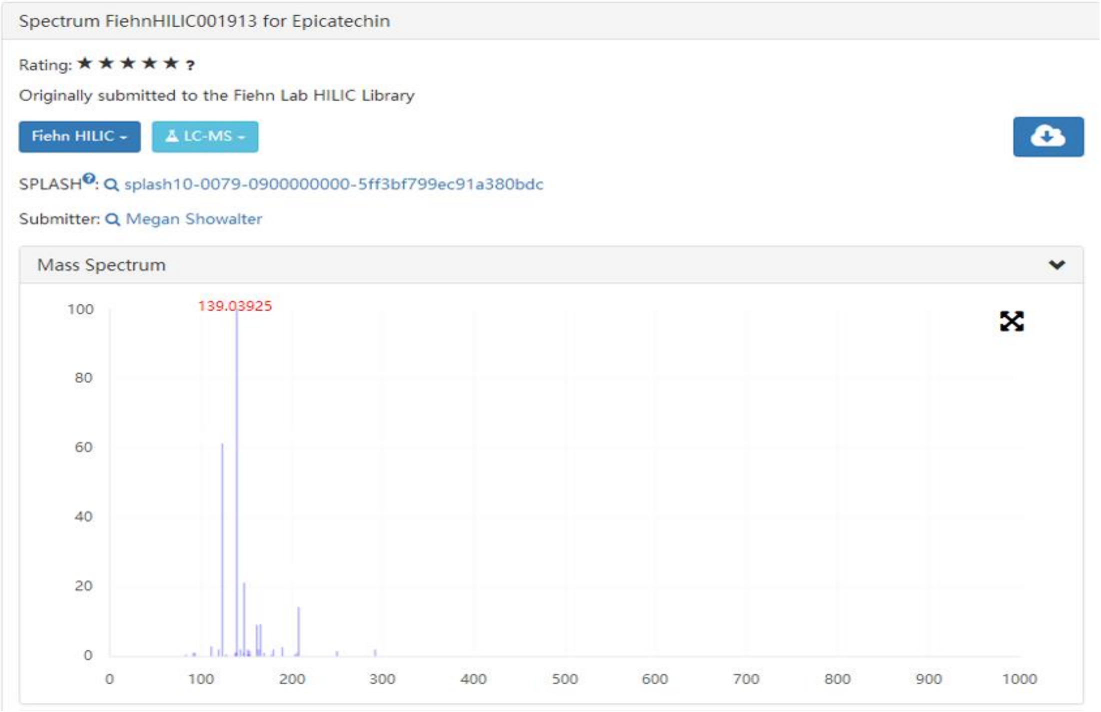

The MS response (4.97 min, m/z 291) of phenolic extracts from unfermented barley (WH-1) and fermented barley (WH-3) were further obtained as follows:

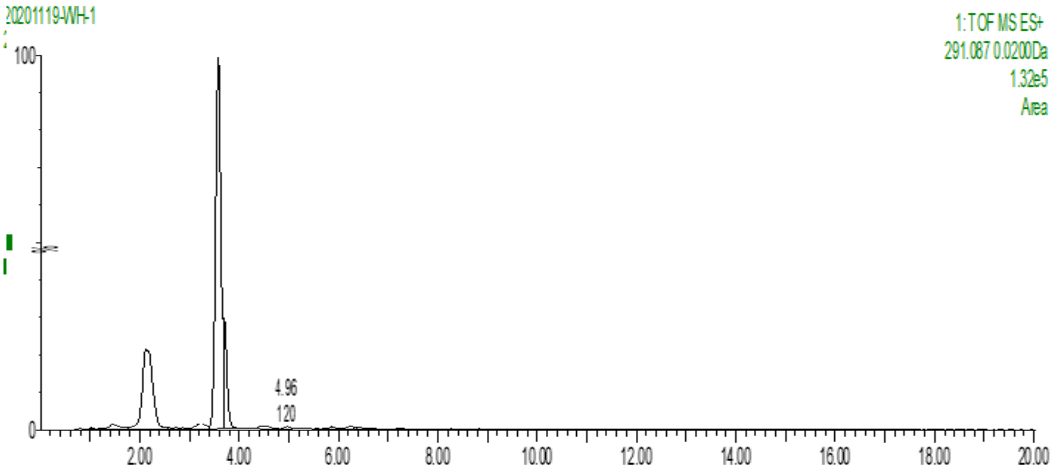

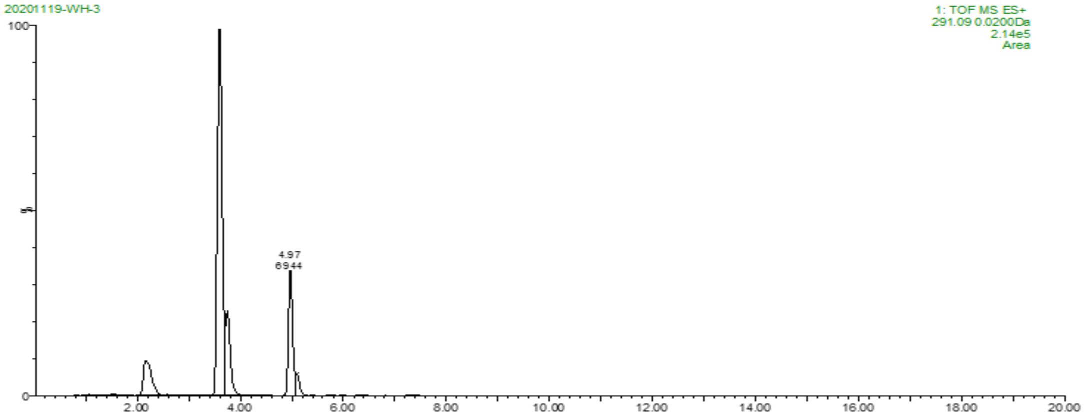

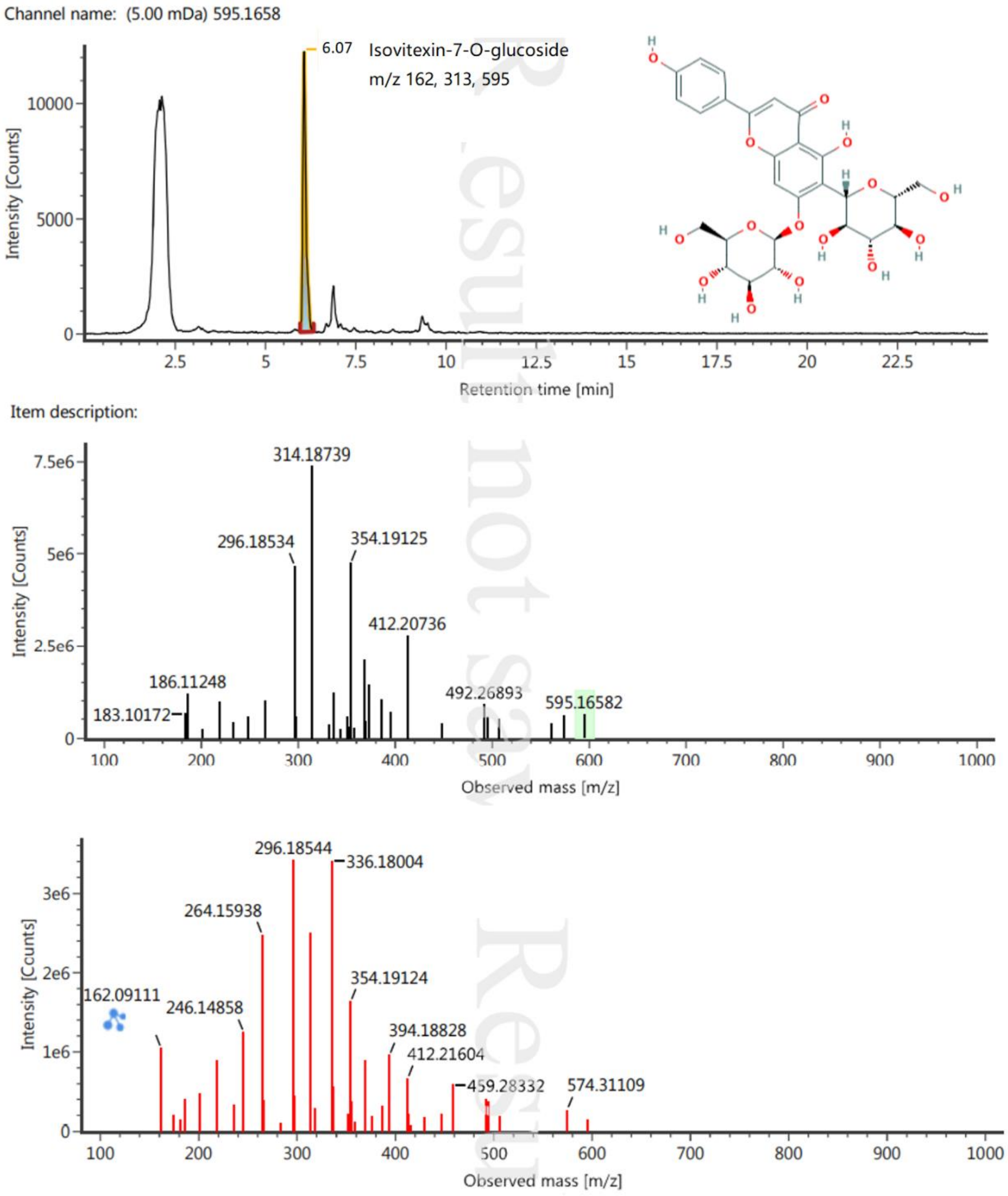

.	Expected m/z	Status	Observed m/z	Mass error (ppm)	Mass error (mDa)	Detector counts	Observed RT (min)	Formula	Observed ion ratio
1	162.08866	Theoretical	162.09111	15.11	2.4	20968	6.01	C7H14O4	
2	236.12544	Theoretical	236.12723	7.59	1.8	7933	6.05	C10H20O6	
3	283.06010	Theoretical	283.06003	-0.26	-0.1	2562	6.08	C16H11O5	
4	313.07066	Theoretical	313.07019	-1.51	-0.5	2785	6.08	C17H13O6	
5	337.07066	Theoretical	337.07006	-1.79	-0.6	2308	6.08	C19H13O6	
6	415.10236	Theoretical	415.10197	-0.93	-0.4	2042	6.08	C21H19O9	

Supplementary Fig. 6 MS of phenolic compound Isovitexin-7-O-glucoside (Saponarin). The mother ion (595 m/z) and fragment ions (162, 313 m/z) obtained were consistent with https://mona.fiehnlab.ucdavis.edu/spectra/display/PS086706, which was as follows:

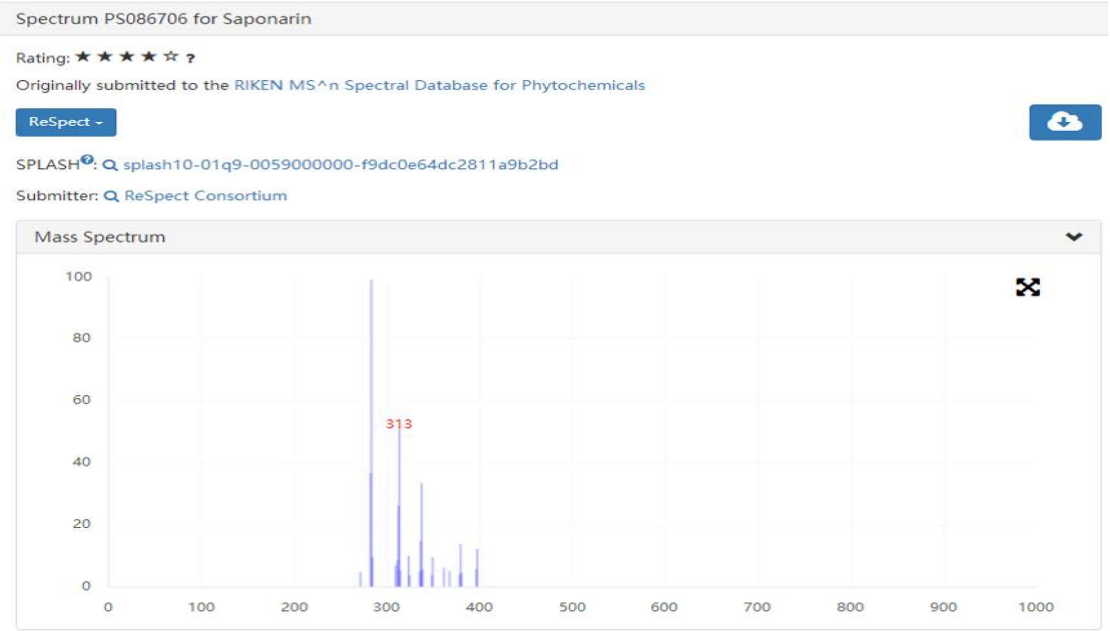

The MS response (6.07 min, m/z 595) of phenolic extracts from unfermented barley (WH-1) and fermented barley (WH-3) were further obtained as follows:

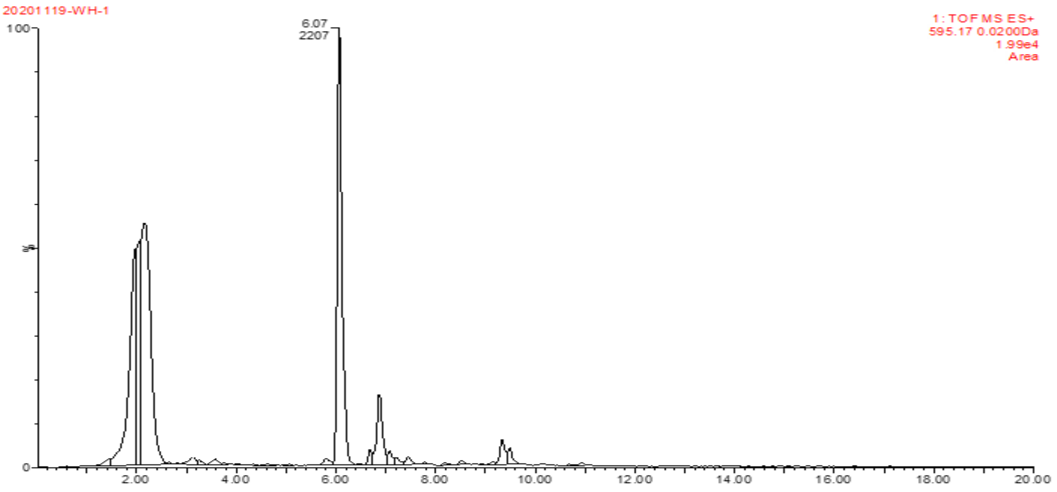

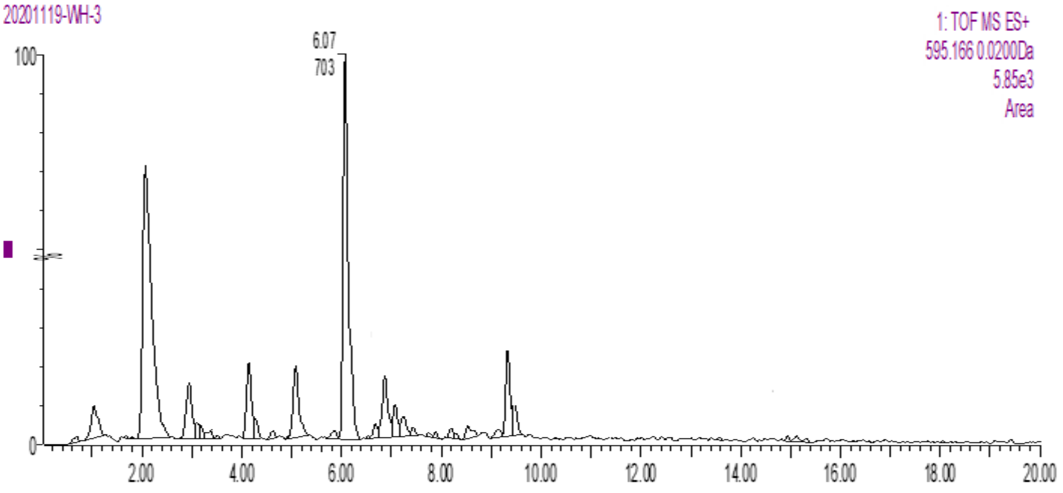

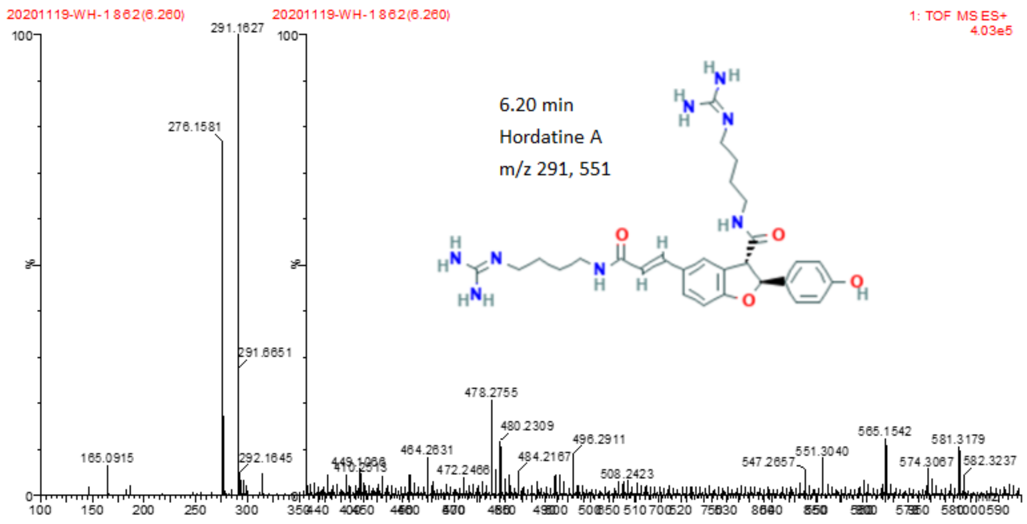

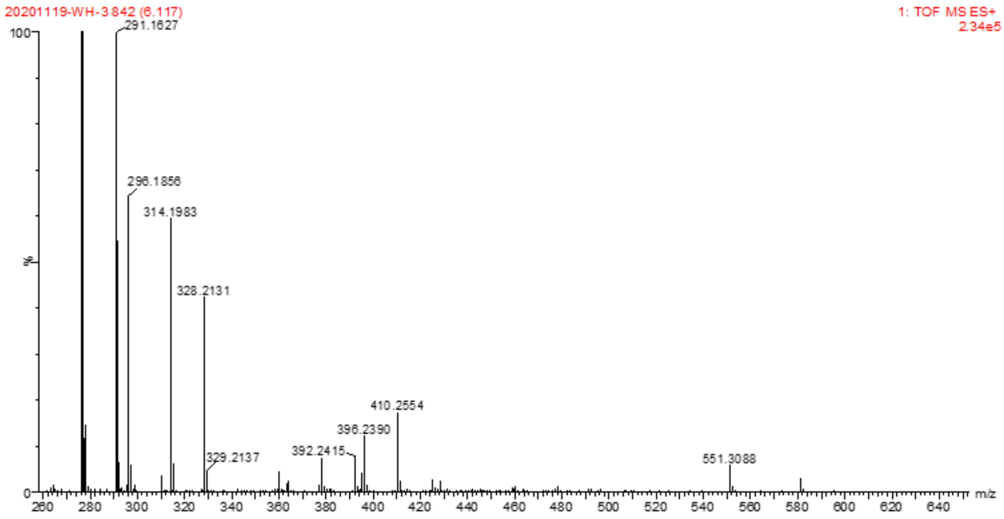

Supplementary Fig. 7 MS of phenolic compound Hordatine A. The mother ion (551 m/z) and fragment ions (291 m/z) obtained were consistent with reports by Krupa-Małkiewicz et al., (2019) and Pihlava (2014).

Krupa-Małkiewicz, M., Oszmiański, J., Lachowicz, S., Szczepanek, M., Jaśkiewicz, B., Pachnowska, K., & Ochmian, I. (2019). Effect of nanosilver (nAg) on disinfection, growth, and chemical composition of young barley leaves under in vitro conditions. *Journal of Integrative Agriculture*, *18*, 1871–1881. https://doi.org/10.1016/S2095-3119(18)62146-X.

Pihlava, J. M. (2014). Identification of hordatines and other phenolamides in barley (*Hordeum vulgare*) and beer by UPLC-QTOF-MS. *Journal of Cereal Science*, *60*, 645–652. http://dx.doi.org/10.1016/j.jcs.2014.07.002.

The MS response (6.20 min, m/z 551) of phenolic extracts from unfermented barley (WH-1) and fermented barley (WH-3) were further obtained as follows:

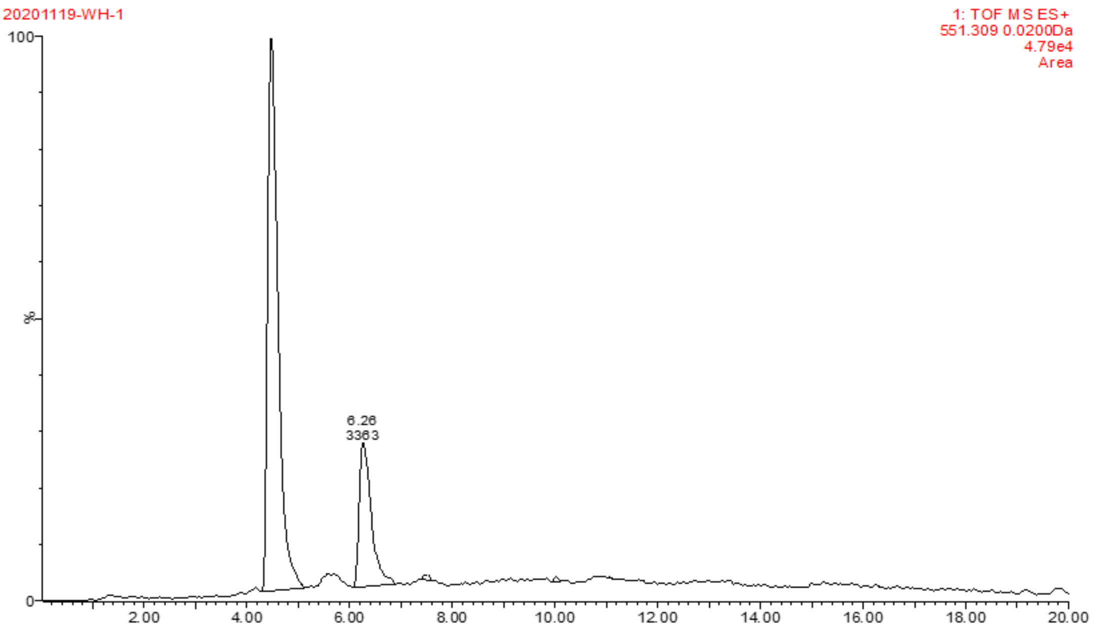

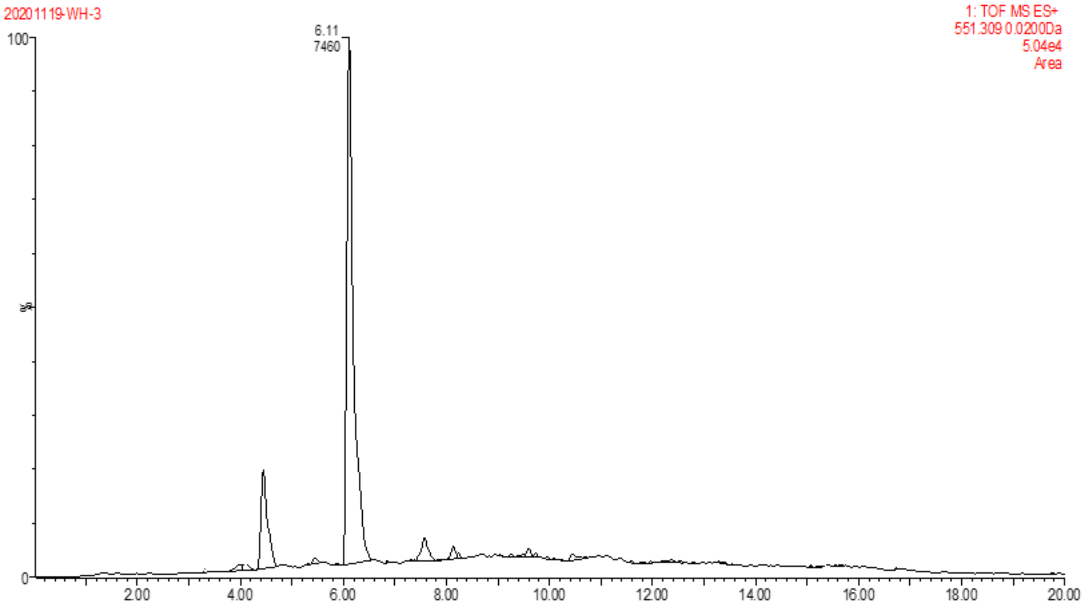

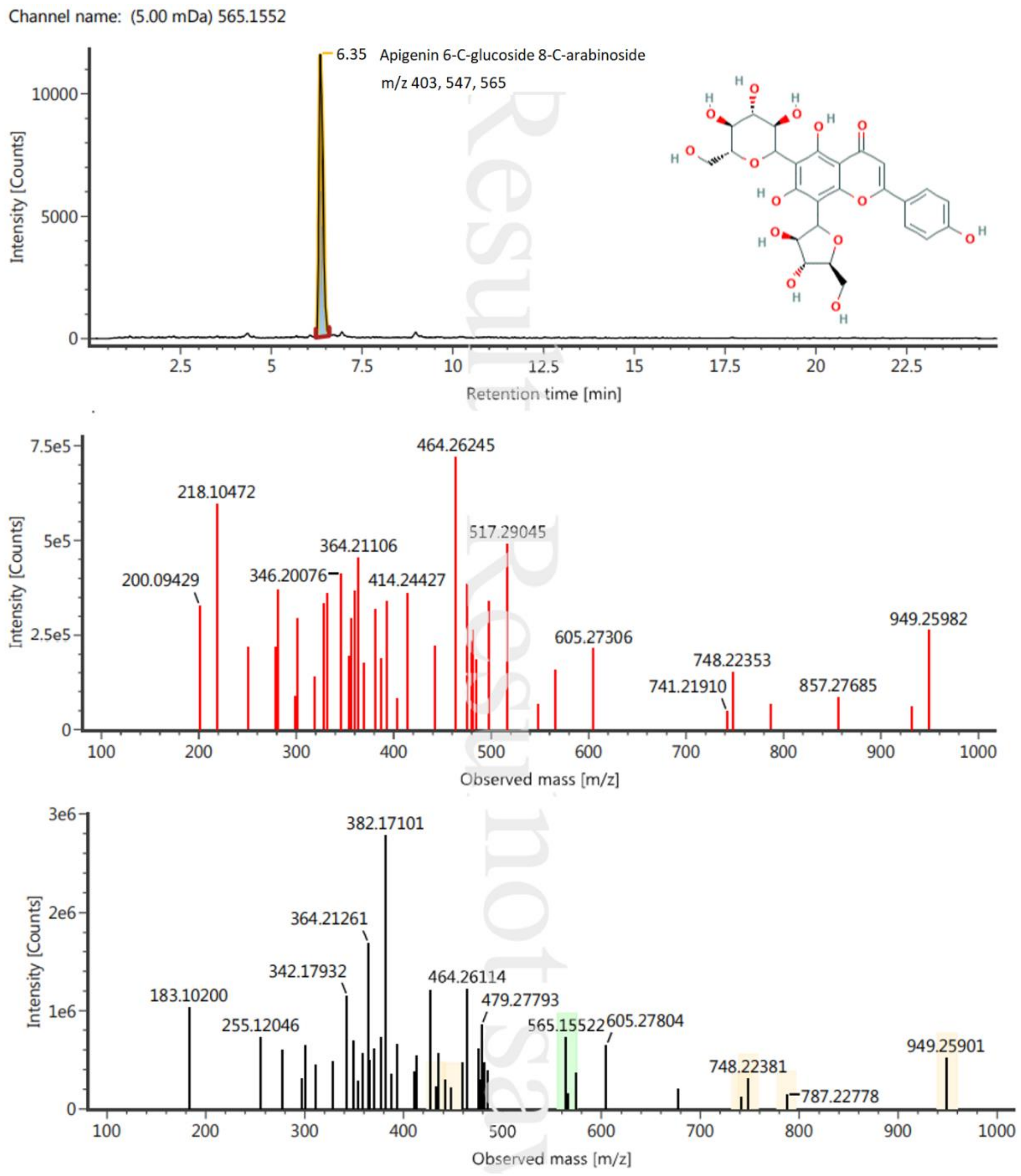

.	Expected m/z	Status	Observed m/z	Mass error (ppm)	Mass error (mDa)	Detector counts	Observed RT (min)	Formula	Observed ion ratio
1	271.06060	Not found							
2	299.05501	Theoretical	299.05423	-2.64	-0.8	2377	6.31	C16H11O6	
3	403.13874	Theoretical	403.13747	-3.16	-1.3	2542	6.36	C21H23O8	
4	547.14462	Theoretical	547.14450	-0.22	-0.1	2443	6.37	C26H27O13	

Supplementary Fig. 8 MS of phenolic compound Apigenin 6-C-glucoside 8-C-arabinoside. The mother ion (565 m/z) and fragment ions (403, 547 m/z) obtained were consistent with https://mona.fiehnlab.ucdavis.edu/spectra/display/PR310990, which was as follows:

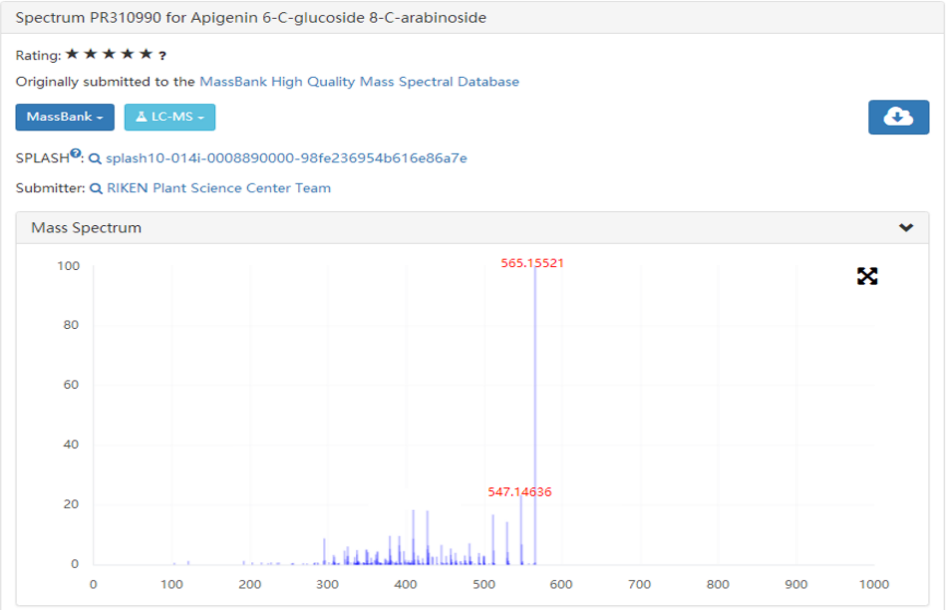

The MS response (6.35 min, m/z 565) of phenolic extracts from unfermented barley (WH-1) and fermented barley (WH-3) were further obtained as follows:

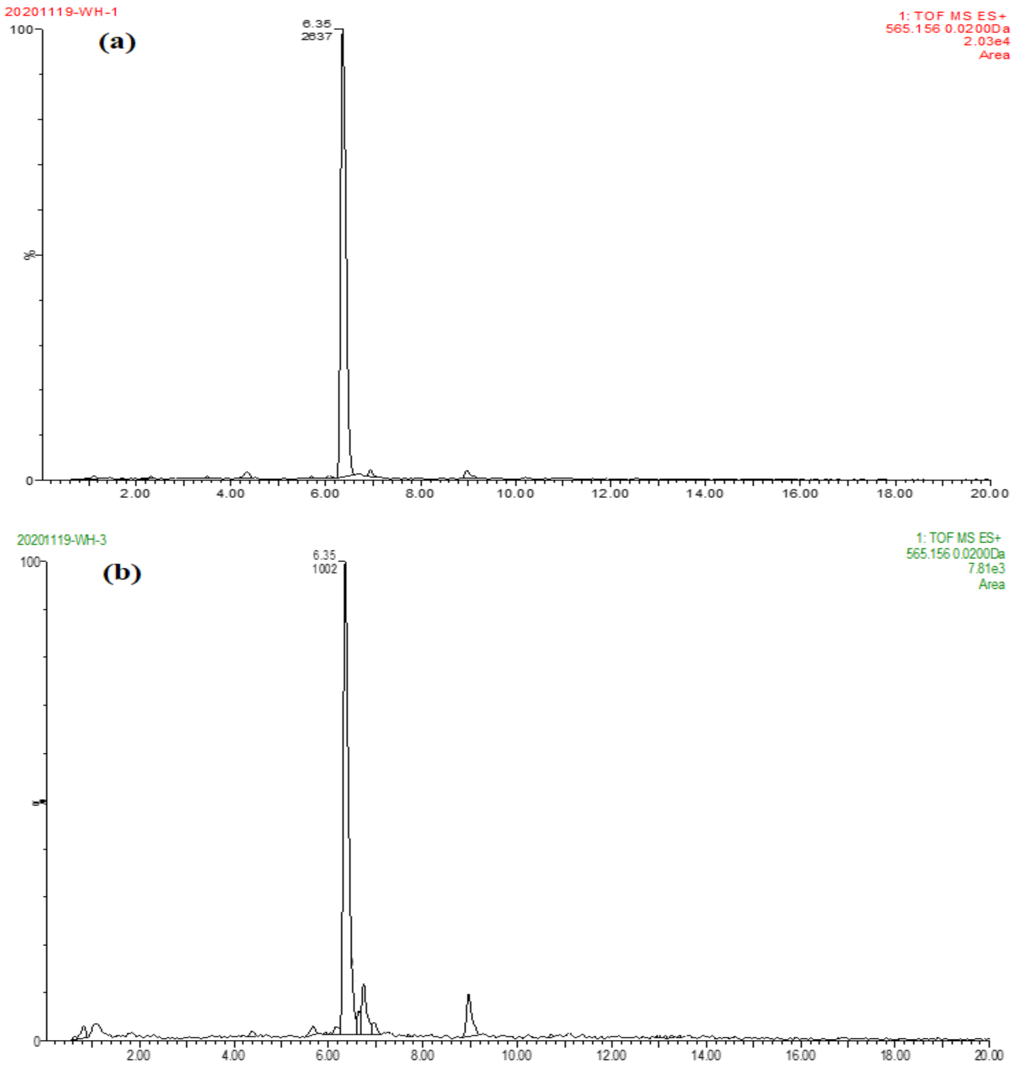

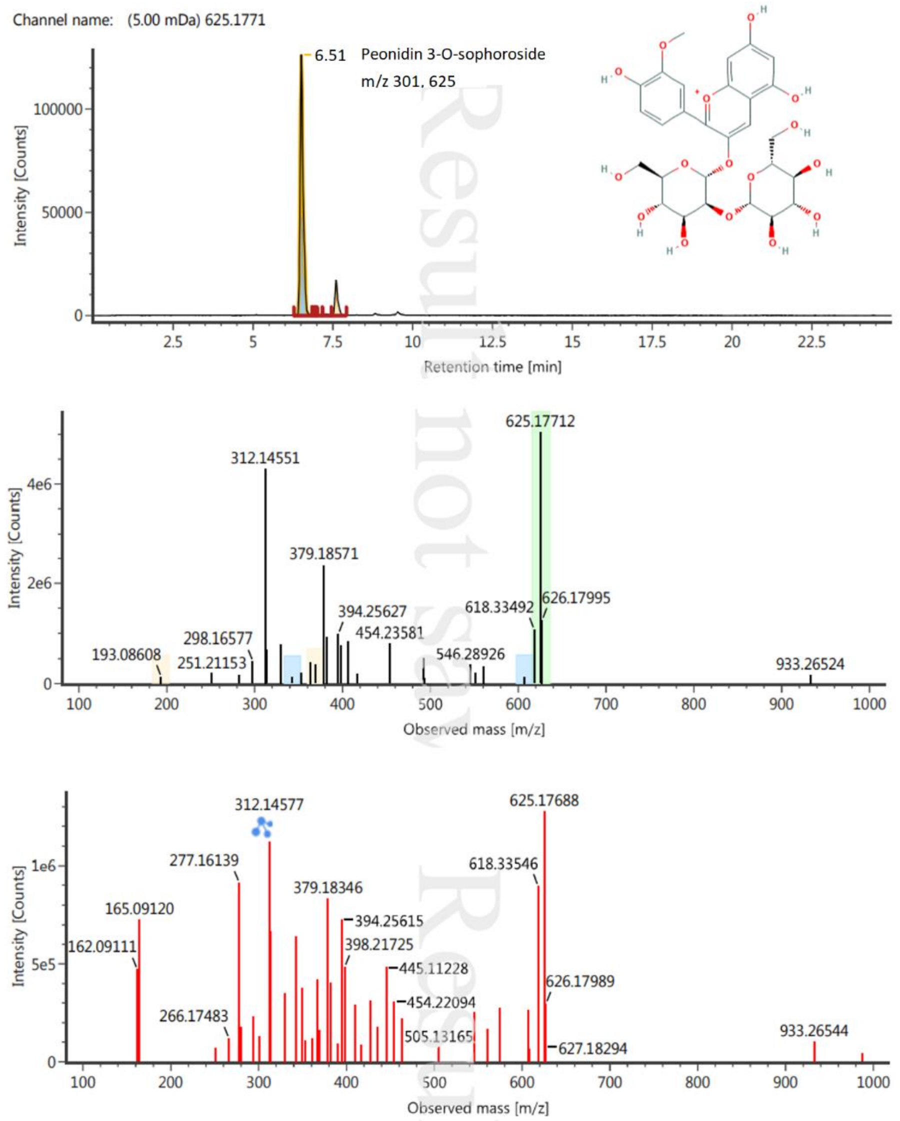

Peonidin 3-O-sophoroside

.	Expected m/z	Status	Observed m/z	Mass error (ppm)	Mass error (mDa)	Detector counts	Observed RT (min)	Formula	Observed ion ratio
1	294.13092	Theoretical	294.12921	-5.81	-1.7	6215	6.47	C12H22O8	
2	301.07066	Theoretical	301.07015	-1.70	-0.5	3517	6.52	C16H13O6	
3	301.07090	Found	301.07015	-2.49	-0.7	3517	6.52		
4	312.14148	Theoretical	312.14577	13.73	4.3	35211	6.47	C12H24O9	
5	313.07066	Theoretical	313.07085	0.59	0.2	21385	6.52	C17H13O6	
6	343.08123	Theoretical	343.08113	-0.30	-0.1	21215	6.52	C18H15O7	
7	367.08123	Theoretical	367.08060	-1.72	-0.6	14677	6.52	C20H15O7	
8	379.08123	Theoretical	379.08096	-0.71	-0.3	2647	6.50	C21H15O7	
9	391.08123	Theoretical	391.07992	-3.34	-1.3	2853	6.52	C22H15O7	
10	397.09179	Theoretical	397.09112	-1.71	-0.7	9257	6.52	C21H17O8	
11	409.09179	Theoretical	409.09133	-1.14	-0.5	9181	6.52	C22H17O8	
12	416.14657	Theoretical	416.15126	11.28	4.7	2679	6.58	C22H24O8	
13	427.10236	Theoretical	427.10173	-1.48	-0.6	12277	6.52	C22H19O9	
14	445.11292	Theoretical	445.11228	-1.44	-0.6	18914	6.52	C22H21O10	
15	463.12349	Theoretical	463.12291	-1.26	-0.6	7483	6.52	C22H23O11	
16	505.13405	Theoretical	505.13165	-4.75	-2.4	2612	6.52	C24H25O12	
17	607.16575	Theoretical	607.16513	-1.02	-0.6	12714	6.52	C28H31O15	

Supplementary Fig. 9 MS of phenolic compound Peonidin 3-O-sophoroside. The mother ion (625 m/z) and fragment ion (301 m/z) obtained were consistent with https://mona.fiehnlab.ucdavis.edu/spectra/display/PM008902, which was as follows:

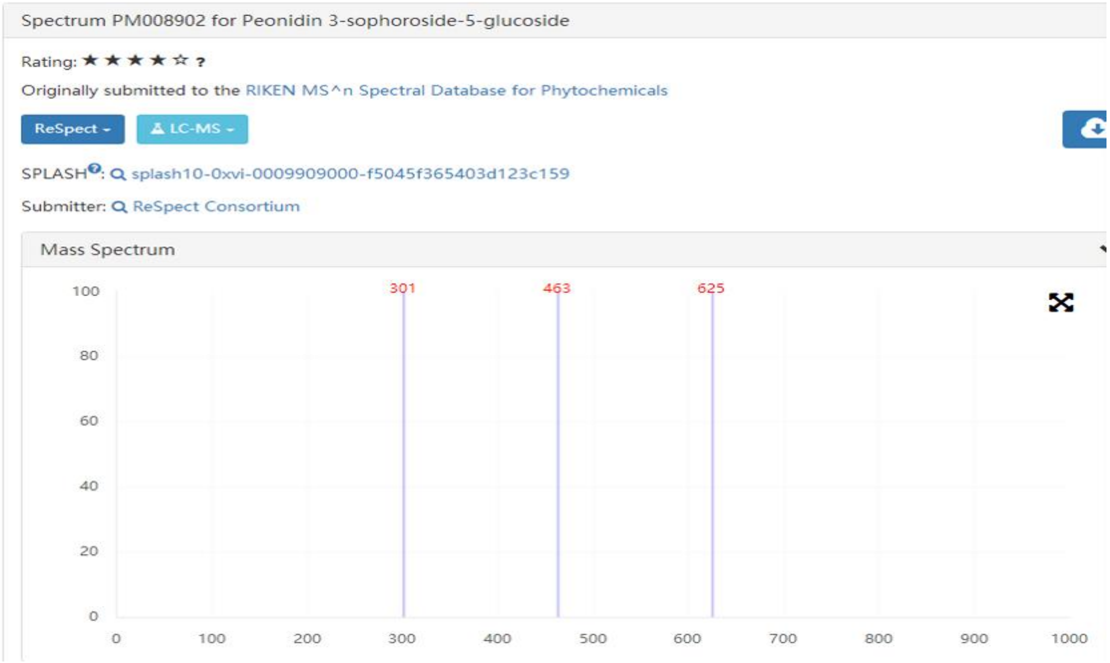

The MS response (6.51 min, m/z 625) of phenolic extracts from unfermented barley (WH-1) and fermented barley (WH-3) were further obtained as follows:

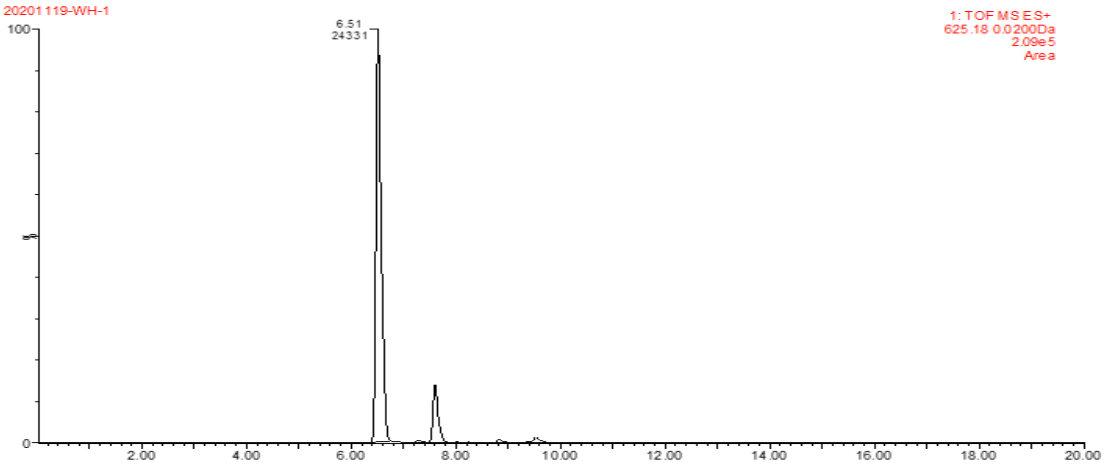

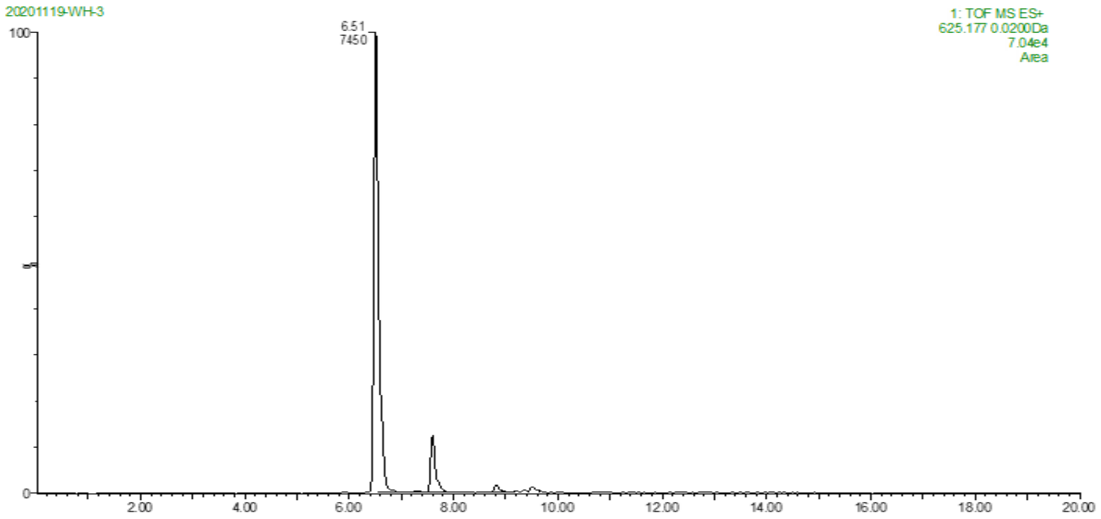

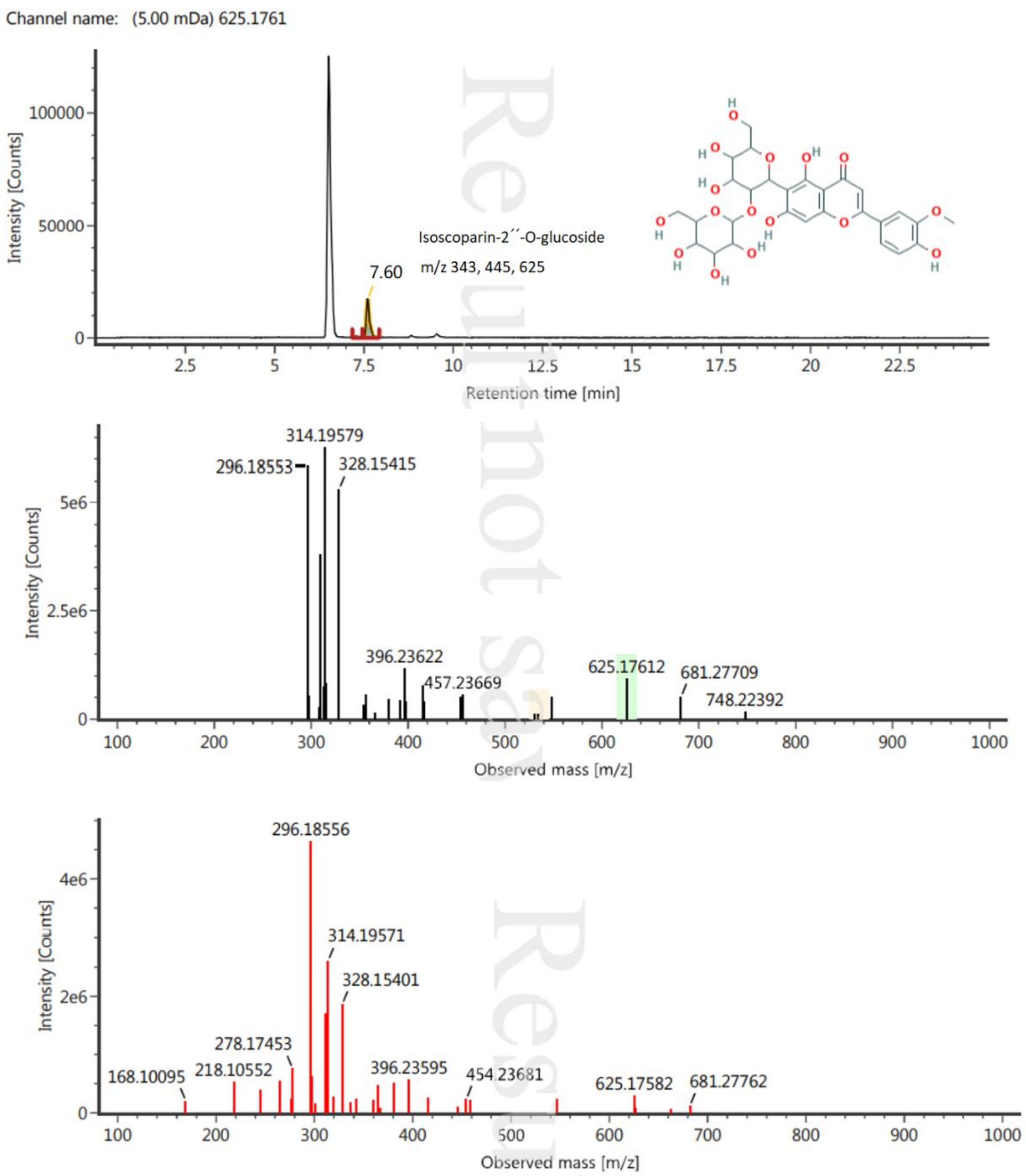

.	Expected m/z	Status	Observed m/z	Mass error (ppm)	Mass error (mDa)	Detector counts	Observed RT (min)	Formula	Observed ion ratio
1	313.07066	Theoretical	313.07068	0.04	0.0	3016	7.61	C17H13O6	
2	317.06610	Not found							
3	343.08123	Theoretical	343.08084	-1.14	-0.4	6682	7.60	C18H15O7	
4	367.08123	Theoretical	367.08022	-2.74	-1.0	2168	7.60	C20H15O7	
5	445.11292	Theoretical	445.11014	-6.26	-2.8	2970	7.59	C22H21O10	

Supplementary Fig. 10 MS of phenolic compound Isoscoparin-2´´-O-glucoside. The mother ion (625 m/z) and fragment ions (343, 445 m/z) obtained were consistent with https://mona.fiehnlab.ucdavis.edu/spectra/display/PR310982, which was as follows:

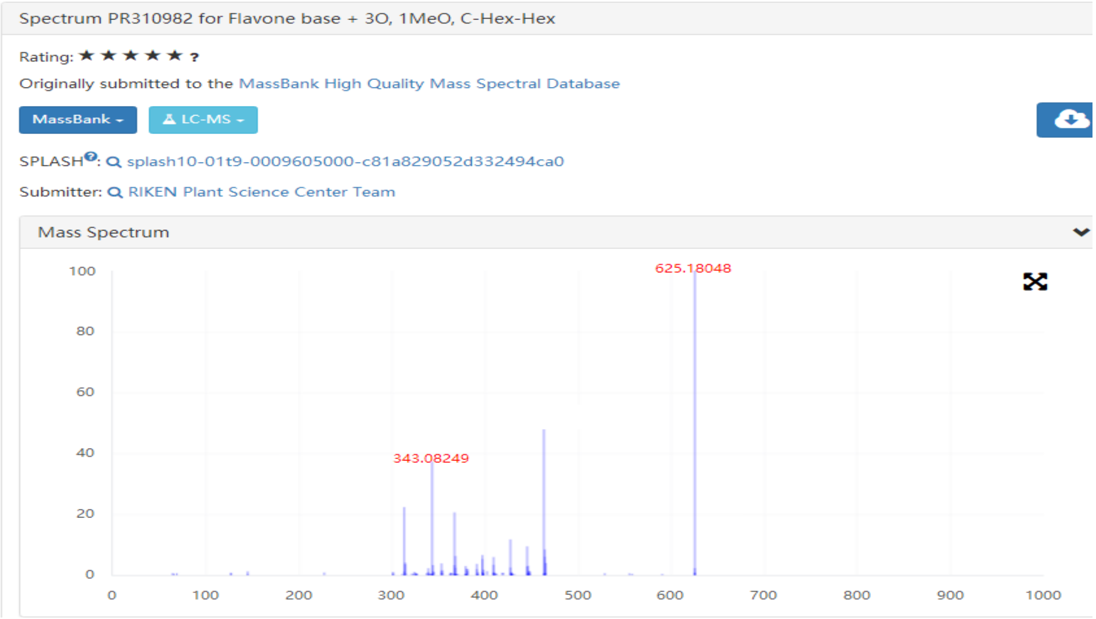

The MS response (7.60 min, m/z 625) of phenolic extracts from unfermented barley (WH-1) and fermented barley (WH-3) were further obtained as follows:

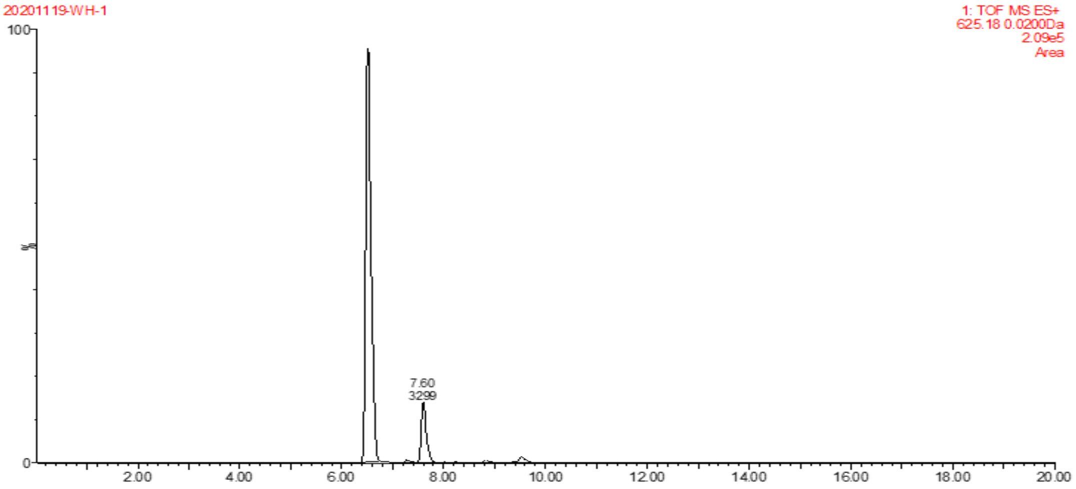

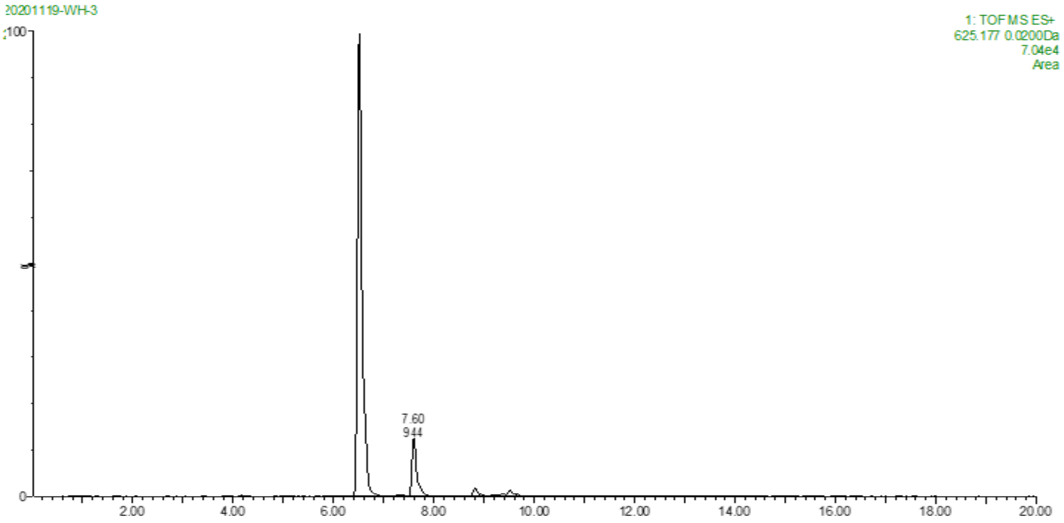

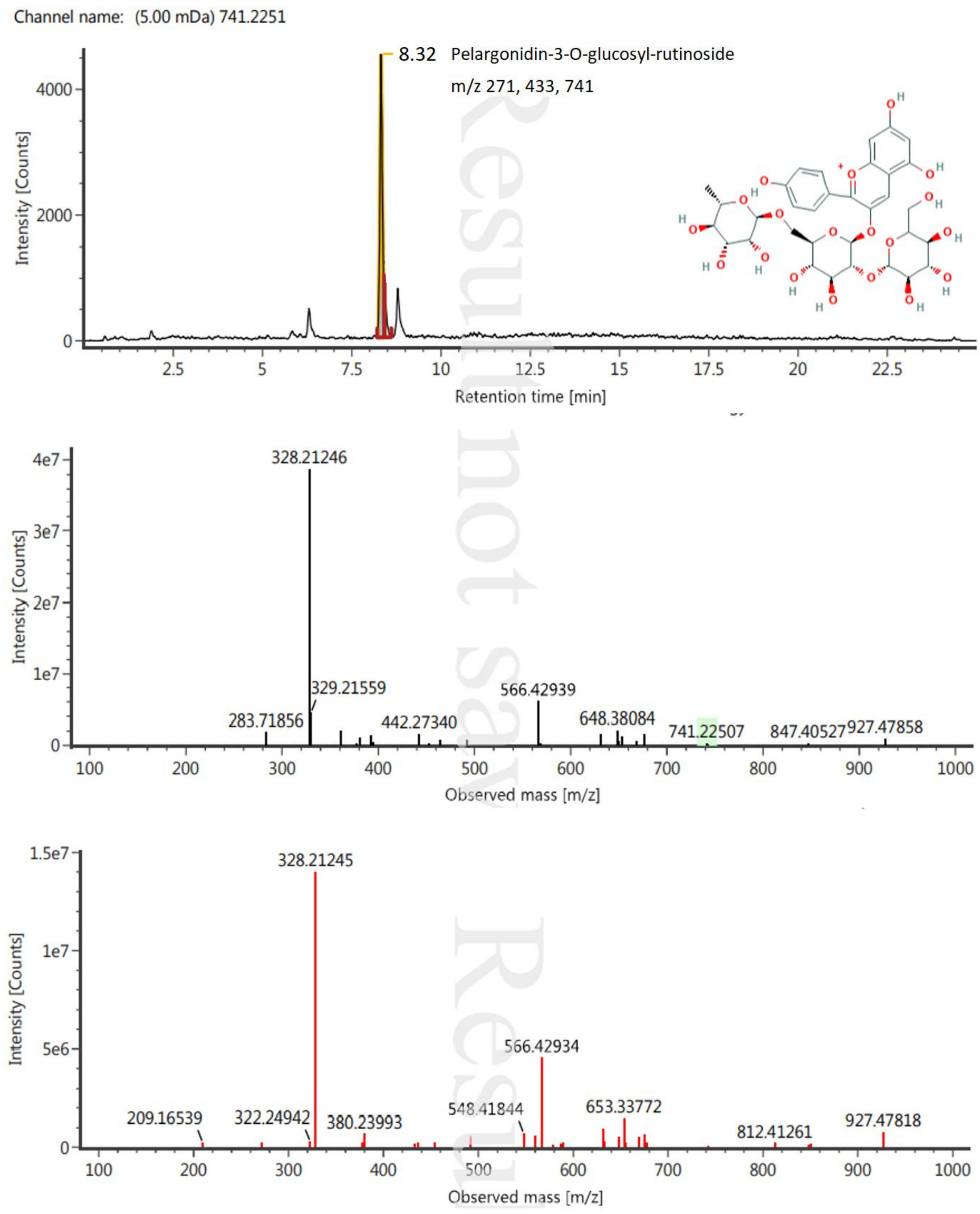

.	Expected m/z	Status	Observed m/z	Mass error (ppm)	Mass error (mDa)	Detector counts	Observed RT (min)	Formula	Observed ion ratio
1	271.06010	Theoretical	271.06081	2.63	0.7	5769	8.32	C15H11O5	
2	271.06060	Found	271.06081	0.79	0.2	5769	8.32		
3	433.11292	Theoretical	433.11304	0.27	0.1	5220	8.32	C21H21O10	
4	579.17083	Theoretical	579.17038	-0.78	-0.4	4062	8.33	C27H31O14	

Supplementary Fig. 11 MS of phenolic compound Pelargonidin-3-O-glucosyl-rutinoside. The mother ion (741 m/z) and fragment ions (271, 433 m/z) obtained were consistent with https://mona.fiehnlab.ucdavis.edu/spectra/display/PR040162, which was as follows:

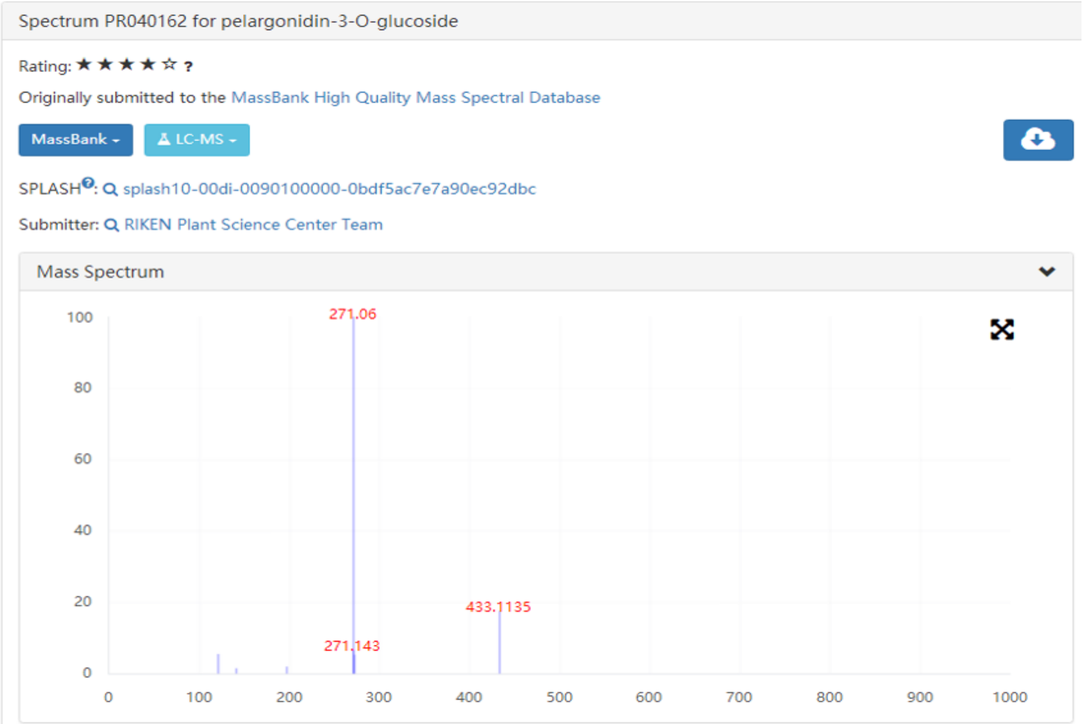

The MS response (8.32 min, m/z 741) of phenolic extracts from unfermented barley (WH-1) and fermented barley (WH-3) were further obtained as follows:

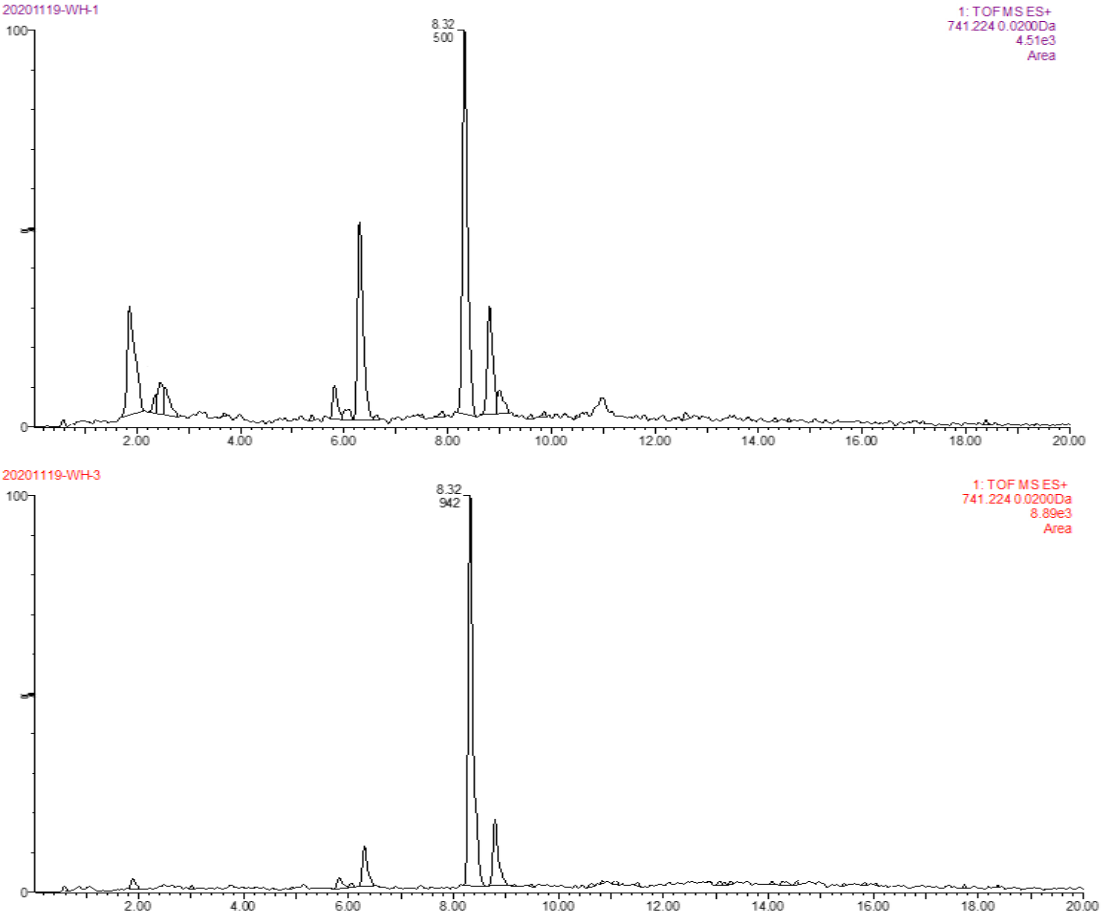

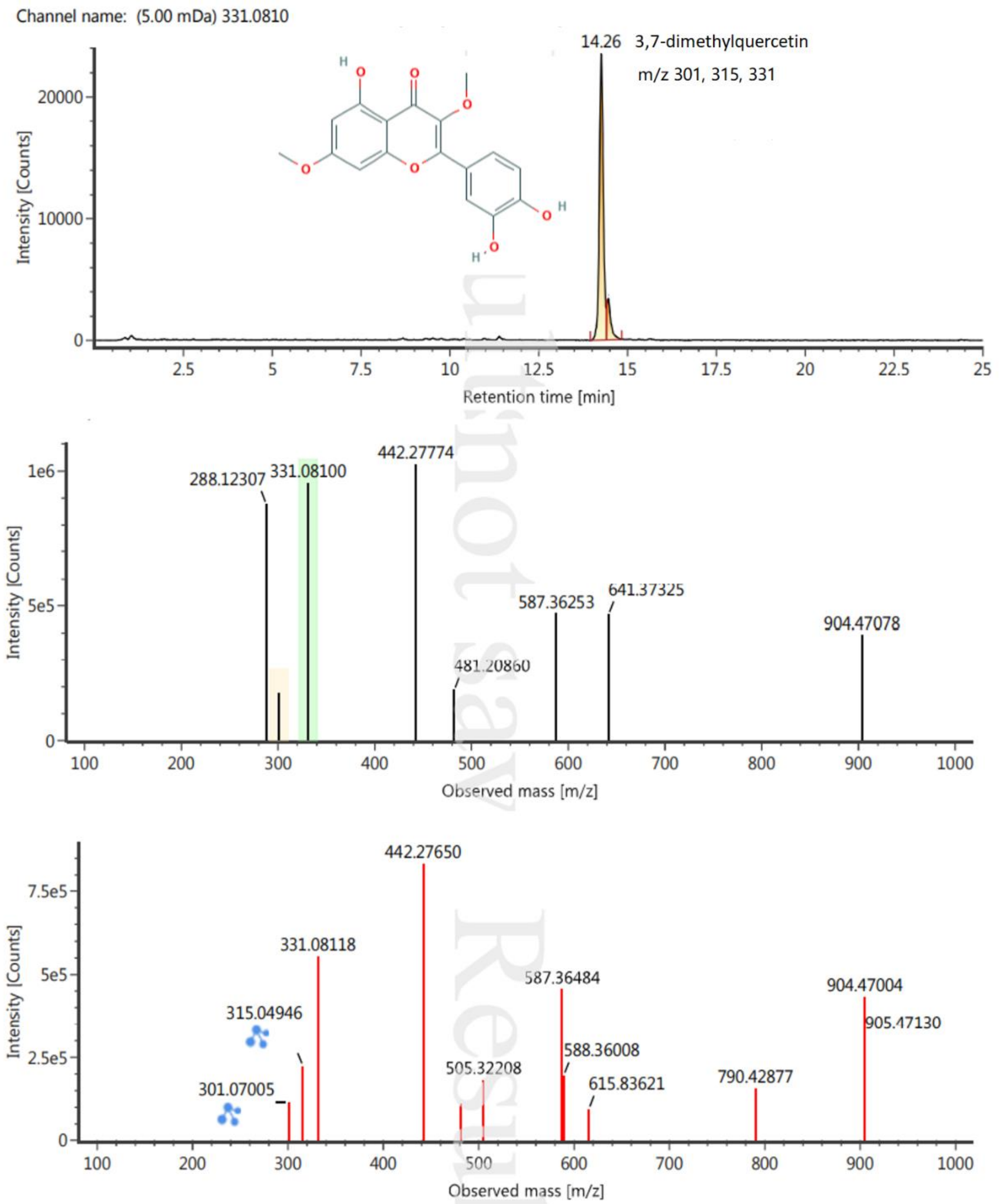

.	Expected m/z	Status	Observed m/z	Mass error (ppm)	Mass error (mDa)	Detector counts	Observed RT (min)	Formula	Observed ion ratio
1	301.07066	Theoretical	301.07005	-2.05	-0.6	3091	14.32	C16H13O6	
2	315.04993	Theoretical	315.04946	-1.48	-0.5	6222	14.26	C16H11O7	

Supplementary Fig. 12 MS of phenolic compound 3, 7-Dimethylquercetin. The mother ion (331 m/z) and fragment ions (301, 315 m/z) obtained were consistent with https://mona.fiehnlab.ucdavis.edu/spectra/display/CCMSLIB00000081737, which was as follows:

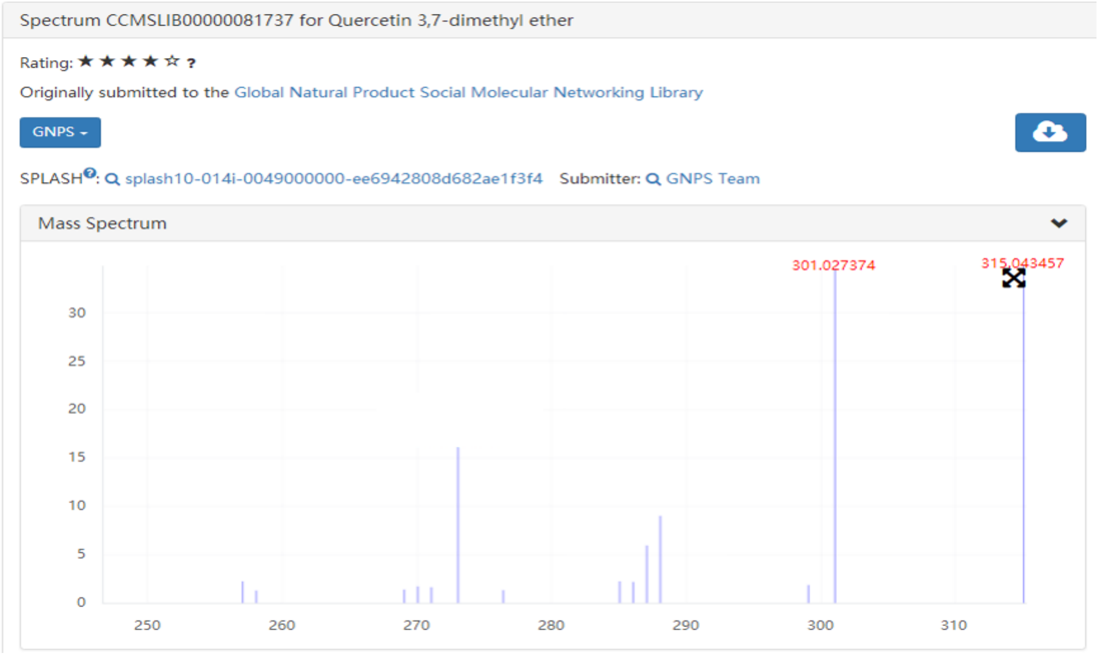

The MS response (14.26 min, m/z 331) of phenolic extracts from unfermented barley (WH-1) and fermented barley (WH-3) were further obtained as follows:

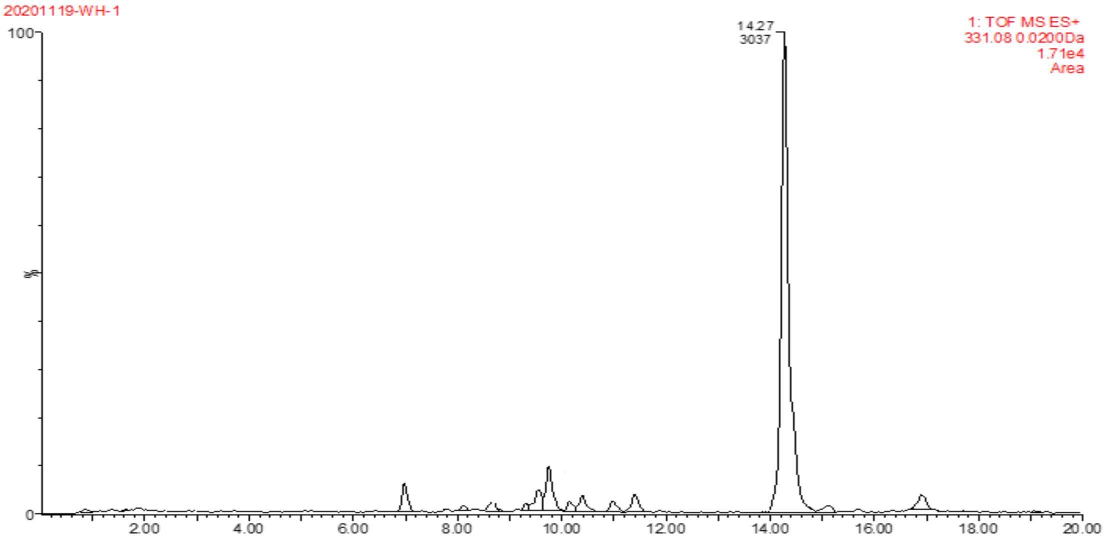

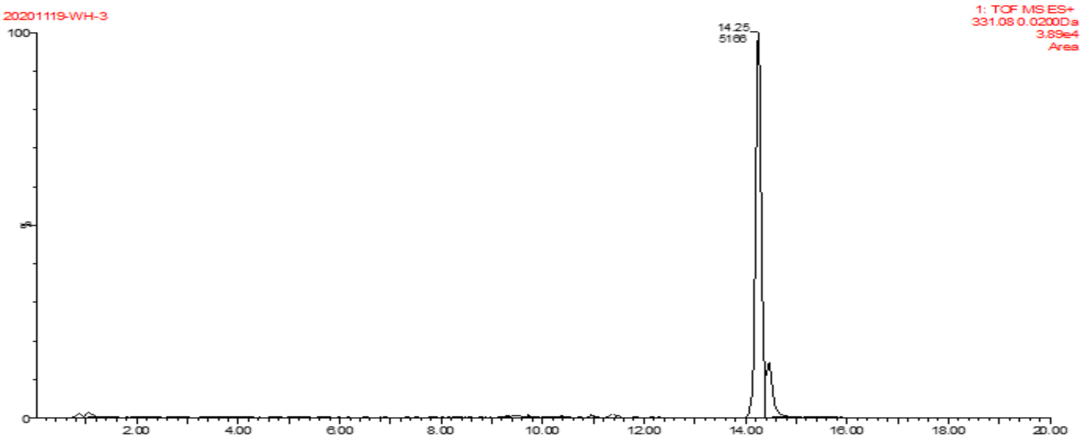

Supplement: Supplementary file 1 [file Data_Sheet_1.pdf]
